# Eco-Label Conveys Reliable Information on Fish Stock Health to Seafood Consumers

**DOI:** 10.1371/journal.pone.0043765

**Published:** 2012-08-21

**Authors:** Nicolás L. Gutiérrez, Sarah R. Valencia, Trevor A. Branch, David J. Agnew, Julia K. Baum, Patricia L. Bianchi, Jorge Cornejo-Donoso, Christopher Costello, Omar Defeo, Timothy E. Essington, Ray Hilborn, Daniel D. Hoggarth, Ashley E. Larsen, Chris Ninnes, Keith Sainsbury, Rebecca L. Selden, Seeta Sistla, Anthony D. M. Smith, Amanda Stern-Pirlot, Sarah J. Teck, James T. Thorson, Nicholas E. Williams

**Affiliations:** 1 Marine Stewardship Council, London, United Kingdom; 2 Bren School of Environmental Science and Management, University of California Santa Barbara, Santa Barbara, California, United States of America; 3 School of Aquatic and Fishery Sciences, University of Washington, Seattle, Washington, United States of America; 4 Department of Biology, University of Victoria, Victoria, British Columbia, Canada; 5 Interdepartmental Graduate Program in Marine Science, Marine Science Institute, University of California Santa Barbara, Santa Barbara, California, United States of America; 6 Universidad Austral de Chile, Centro Trapananda, Coyhaique, Chile; 7 UNDECIMAR, Facultad de Ciencias, Montevideo, Uruguay; 8 Department of Ecology, Evolution and Marine Biology, University of California Santa Barbara, Santa Barbara, California, United States of America; 9 University of Tasmania, Tasmanian Aquaculture & Fisheries Inst, Taroona, Tasmania, Australia; 10 Commonwealth Scientific and Industrial Research Organization, Wealth from Oceans Flagship, Hobart, Tasmania, Australia; 11 Department of Anthropology, University of California Santa Barbara, Santa Barbara, California, United States of America; University of Hamburg, Germany

## Abstract

Concerns over fishing impacts on marine populations and ecosystems have intensified the need to improve ocean management. One increasingly popular market-based instrument for ecological stewardship is the use of certification and eco-labeling programs to highlight sustainable fisheries with low environmental impacts. The Marine Stewardship Council (MSC) is the most prominent of these programs. Despite widespread discussions about the rigor of the MSC standards, no comprehensive analysis of the performance of MSC-certified fish stocks has yet been conducted. We compared status and abundance trends of 45 certified stocks with those of 179 uncertified stocks, finding that 74% of certified fisheries were above biomass levels that would produce maximum sustainable yield, compared with only 44% of uncertified fisheries. On average, the biomass of certified stocks increased by 46% over the past 10 years, whereas uncertified fisheries increased by just 9%. As part of the MSC process, fisheries initially go through a confidential pre-assessment process. When certified fisheries are compared with those that decline to pursue full certification after pre-assessment, certified stocks had much lower mean exploitation rates (67% of the rate producing maximum sustainable yield vs. 92% for those declining to pursue certification), allowing for more sustainable harvesting and in many cases biomass rebuilding. From a consumer’s point of view this means that MSC-certified seafood is 3–5 times less likely to be subject to harmful fishing than uncertified seafood. Thus, MSC-certification accurately identifies healthy fish stocks and conveys reliable information on stock status to seafood consumers.

## Introduction

The global per-capita demand for seafood has reached an all-time high, and is likely to continue to increase [1]. Wild capture seafood harvest has also peaked, and while management measures have led to rebuilding in some fish stocks, one-third of the world’s well-studied fisheries are overfished [1]–[3]. To maintain or enhance wild fish supplies on a sustainable basis management agencies and governments must rebuild fisheries whose stocks are at low biomass and maintain healthy stocks at or above sustainable levels. Fisheries and conservation objectives can be attained by redundancy in management actions, including catch controls, gear modifications, closed areas, and community-based management, depending on local context and specific features [2], [4]. One way of influencing these fishery practices is through market-based approaches such as “eco-labeling” which aim to harness consumer preferences to increase market demand, and often prices, for well-managed fisheries and diminish demand for others [5], [6]. To further this aim, national and global schemes designed to allow consumers to make informed choices when purchasing seafood have proliferated [5]. These efforts include awareness campaigns such as consumer guides produced by Monterey Bay Aquarium, World Wildlife Fund and Greenpeace, the risk of extinction Red List categories of the IUCN, and certification and eco-labeling programs such as the Marine Stewardship Council (MSC) and Friend of the Sea [7].

Unlike some consumer awareness campaigns, certification programs such as the MSC consider a fishery or fish stock, rather than a species, to be the primary unit of certification. This acknowledges variation in harvest practices among fleets and recognizes those that adopt environmentally sound activities [7]. In theory, eco-labels convey information about these improved fishing practices to consumers, who then make choices about what seafood to buy based on this information. Consumer preference can result in increased prices [8] and indirect non-economic benefits for fishers [9], [10] and access to markets looking to exclusively source certified fish products [7], [11]. Moreover, leading supermarkets and restaurant chains recognize that consumers increasingly expect retailers to make responsible purchasing decisions as part of their corporate social responsibility, and may require third-party certification in the products they source. These act as incentives for improvement in uncertified fisheries and for continued stewardship in certified fisheries. The conservation value of eco-labels, however, relies on their ability to convey accurate information to consumers about the sustainability of fisheries.

The Marine Stewardship Council is the most prominent global fisheries eco-label program. It arose from a partnership between the World Wildlife Fund and Unilever in 1996, and has operated as an independent non-profit since 1999. There are currently 132 MSC-certified fisheries and 141 more at different stages of the certification process. MSC-certified seafood covers 10% of the annual global harvest of wild capture fisheries and more than 13,000 products, by far the highest representation of eco-labeled seafood in global markets [7], [8]. The MSC’s rapid growth has stoked debate about what constitutes a sustainable fishery, and the decisions to certify certain fisheries as sustainable have been scrutinized. Recent criticisms have questioned the rigor of the MSC’s certification standards for ecosystem impacts of fisheries that damage habitats or result in high levels of bycatch [12]–[16]. There have been calls to focus certification on small-scale fisheries [12], [16]–[18] based on the perception that they have a lower environmental impact than industrial fisheries [16], despite a paucity of data with which to assess the sustainability of small-scale fisheries. The strongest criticism of the MSC, however, argues that its certification standards fail to accurately identify healthy stocks, with several case studies cited by critics as not being sustainably managed, including Pacific Hake (*Merluccius productus*) and Eastern Bering Sea (EBS) walleye pollock (*Theragra chalcogramma*) [12], [16].

The MSC certifies fisheries as sustainable only if they score highly on each of 3 principles [19]: (1) fishing should be conducted in a way that prevents overfishing (depletion of exploited populations beyond biological limits) through the use of target reference points that should maintain the stock at or above the biomass that produces the maximum sustainable yield (MSY), and overexploited stocks must be demonstrably on a path to recovery; (2) fishing operations must maintain the structure, diversity, function, and productivity of associated ecosystems; and (3) the management system must respect national and international regulations. Fisheries applying for MSC certification first undergo a confidential pre-assessment stage to evaluate their potential for meeting the certification standard. Based on this evaluation, fishing industry groups decide whether to undergo a public full assessment by an independent third party. Fisheries meeting the above standards are certified for five years and undergo annual surveillance audits. Those that meet the standard but are weak in certain areas can be certified if they commit to and demonstrate progress toward meeting agreed conditions on improvement. Thus, fisheries must demonstrate continued adherence to, and improvement in, a variety of aspects of sustainability to maintain their certification status.

The term “sustainable” is difficult to define because it encompasses ecological, social, and economic components. At a basic level, however, a renewable resource must be extracted no faster than the level at which it can replace itself for it to be considered sustainable. If certified fisheries are no better at identifying and responding to low biomass levels than uncertified fisheries, then eco-labeling is unlikely to catalyze widespread improvements in fisheries management [14]. Therefore the decline of some certified stocks has cast doubt on the validity of the information conveyed by the MSC label.

Here we assess the performance of fish stocks against Principle 1 (targeted population status), specifically evaluating the status and harvest levels of fish stocks targeted by MSC-certified fisheries, because recent criticisms of MSC have questioned whether these fisheries actually target healthy stocks [12], [16]. While all three principles are equally weighted criteria in the MSC’s assessment process, Principles 2 and 3 address effects of fishing whose impacts can be difficult to measure directly. Thus assessing the performance of certified vs. uncertified fish stocks is a critical first step in evaluating the effectiveness of the MSC’s certification standards.

## Methods

### Data

We compiled time series of catch data and model estimates of biomass and fishing mortality rates for all stocks where the above information was available (45 certified stocks, Table S1, and 179 uncertified stocks, Table S2). Certified stocks managed under different schemes than single-species MSY (e.g., salmon and invertebrates) or without biomass time series and thus qualitatively assessed under a risk-based framework [19] (e.g., small-scale and data-deficient fisheries) were excluded from our analysis. However, given that some stocks are targeted by multiple certified fisheries, analyzed stocks represented 62% (*n* = 82) of the total certified fisheries (*n* = 133) and 85% of the certified landings (4.5 million tons).

The majority of data was sourced from the RAM Legacy Stock Assessment Database (20), which represents the largest global stock assessment database currently available, but status was updated if newer stock assessments were available (Table S1 and Table S2). For each stock we recorded the biomass (*B*_MSY_) and exploitation rate (*u*_MSY_) or fishing mortality (*F*_MSY_) that results in MSY from published stock assessments. When estimates of one or both reference points were not available, they were estimated by fitting Schaefer surplus production models [2], [20] to time series of biomass estimates using a maximum likelihood approach in AD Model Builder [21]. Table S1 includes the method used for each certified stock, and Table S2 lists the method for each uncertified stock. Finally, we collected time series of biomass estimates from stock assessments in order to compare long-term trends in biomass relative to target *B*_MSY_ for certified and uncertified stocks.

The confidential MSC pre-assessment process screens out many applicants that are unlikely to achieve full certification. Since 1997, 447 fisheries have applied for pre-assessment. Of these fisheries, 55% were not recommended to enter full assessment due either to major management weaknesses (35% of the 447 fisheries) or because they had low biomass, high exploitation rates, or insufficient information to judge stock status (20% of the 447 fisheries) [22]. Of the fisheries not recommended to enter full assessment, 25 stocks for which information was available were included in our analysis (Text S1). Thus our analysis compared certified stocks with both uncertified stocks and the 25 stocks (counted among the 179) that a pre-assessment had suggested would fail the Principle 1 standard. Due to confidentiality, details about the identities of these stocks cannot be released.

### Definitions and Analysis

According to international agreements and many national laws, fish stocks should be maintained at or rebuilt to a size that can support MSY [23], [24]. This biomass, denoted *B*_MSY_, is typically 20–50% of the average population biomass in the absence of fishing [25]. The corresponding annual exploitation rate (*u*, catch divided by total biomass) that stabilizes the stock around *B*_MSY_ is *u*_MSY._ We follow international convention by using MSY-based reference points, which remain the most widely used method of assessing whether stocks are overfished or not. For example, the UN Convention on the Law of the Sea [23] requires countries to rebuild to MSY levels, and the United Nations Fish Stock Agreement [24] specifies *F*_MSY_ as a reference point. We acknowledge that there is some debate about whether MSY reference points are an appropriate target for sustainability, and that alternative methods may result in differing estimates of *B*_MSY_ and *F*_MSY_, but these are larger issues than can be addressed in this paper.

For a fishery to be MSC-certified, biomass should be at or fluctuating around *B*_MSY_, or if consistently below *B*_MSY_, should be under a rebuilding plan (i.e., *F*<*F*_MSY_) that will lead to recovery of the stock in the near future [19]. There is also a minimum level of biomass, or limit reference point, below which stocks are considered overfished and certification cannot be obtained irrespective of any rebuilding plan. Limit reference points must be set such that if a stock is maintained above these, there is a very low risk of impaired recruitment. These MSY reference points are currently the most informative benchmark with which to assess status across a global sample of fish stocks [2], [20].

We compared the biomass status and exploitation rate in relation to MSY targets of certified, uncertified, and non-recommended fish stocks by plotting *B*/*B*_MSY_ vs. *u*/*u*_MSY_ or *F*/*F*_MSY_ and using kernel density smoothing functions to describe the probability of occurrence in each quadrant. To determine whether *B*/*B*_MSY_ and *u*/*u*_MSY_ were significantly different between groups we used re-sampling inference (100,000 times without replacement), which allows us to assess how often a difference of the observed magnitude or larger would arise by chance. We also estimated the proportion of stocks in each group that met or exceeded biomass and harvest targets and that were below 0.5*B_MSY_* (as a proxy for the U.S. legal definition of overfished, or the point below which recruitment can be impaired for certain stocks; [26]) and above a more conservative target of 1.3*B_MSY_* [27]. The long-term performance of certified and uncertified stocks in relation to *B_MSY_* was assessed using time series data from 1970 to the present (available for 165 uncertified stocks and 31 certified stocks). Using an autoregressive model we tested for differences in the conditional mean of *B*/*B*_MSY_ between certified and uncertified stocks over time. The model structure was selected through Akaike’s Information Criteria (AIC) and model parameters were estimated using Ordinary Least Squares.

## Results and Discussion

### Statuses of Certified and Uncertified Stocks

**Figure 1:**
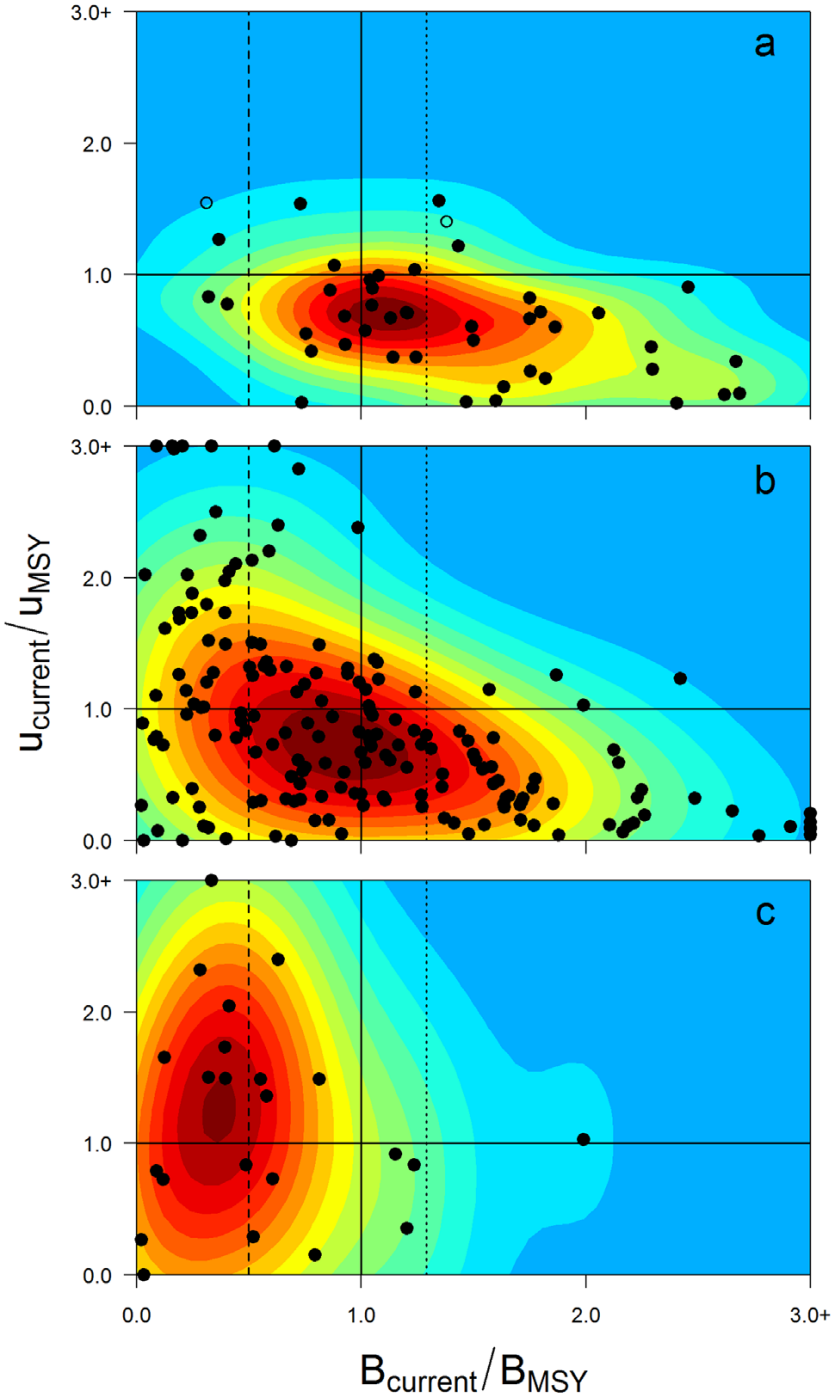
Sustainability of certified and uncertified seafood. Current (i.e., most recent year with available information) biomass and exploitation rate for (**A**) individual certified (*n* = 45); (**B**) all uncertified (*n* = 179); and (**C**) uncertified stocks that went through pre-assessment and were not recommended for certification (*n* = 25). Data are scaled relative to *B*MSY and *u*MSY or *F*MSY (the biomass and exploitation rates or fishing mortality rates that produce maximum sustainable yield). Contour colors show probability of occurrence (red indicates the highest probability and blue the lowest). Vertical and horizontal solid lines represent reference points common to all fisheries (*B/B*MSY = 1 and *u/u*MSY or *F/F*MSY = 1). Dotted lined represents *B* = 1.3*B*MSY and dashed line *B* = 0.5*B*MSY. Footnote: New assessments for some fish stocks were released while this paper was in press, but this figure was not updated to maintain consistency in year of release.

Our analysis indicates that the ratio of current biomass to the biomass at MSY (*B_current_*/*B*_MSY_) is significantly different between certified and uncertified fisheries (1.25 vs. 0.87; *P*<0.005), and between certified and non-recommended fisheries (1.25 vs. 0.48; *P*<0.005; Table S3 and Table S4). We found 74% of the certified stocks to be currently above sustainable target biomass levels (i.e., *B*_current_>*B*_MSY_; Fig. 1A) (Text S1), compared with 44% of uncertified stocks, and 16% of non-recommended fisheries (Fig. 1B; Table S3). Given that certification assumes harvesting at MSY levels, which will result in biomass fluctuating around *B*_MSY_, we would expect 50% of stocks above *B*_MSY_ and 50% below *B*_MSY_. Thus, our finding that three quarters of stocks targeted by MSC-certified fisheries are above *B*_MSY_ suggests that managers of these stocks are often aiming to ensure biomass is not kept near *B*_MSY_ but above *B*_MSY_. Additionally, 82% of certified stocks had current exploitation rates that are expected to maintain the stocks at *B*_MSY_ or allow for rebuilding to *B*_MSY_ (i.e., *u*_current_<*u*_MSY_) compared with 65% of uncertified stocks and 52% of non-recommended stocks.

### Non-recommended Stocks

An analysis of non-recommended stocks revealed that 52% were below 0.5*B*_MSY_ and 48% had exploitation rates higher than *u*_MSY_ (Fig. 1C). These levels are significantly worse than those for certified stocks (*P*<0.005; Table S3), providing further evidence that the pre-certification process screens out stocks that do not meet internationally recognized standards for stock health. It may seem puzzling that such fisheries would apply for certification in the first place, but this may reflect differing perceptions of what sustainability means, as well as different reasons for pursuing certification. Non-recommended fisheries also show poorer stock status when compared with the global sample of uncertified fisheries. Reasons for such differences may be due to a lack of market incentives to pursue certification for some fisheries targeting healthy stocks [10], or due to weaknesses in other aspects of uncertified fisheries such as management systems or bycatch considerations [22]. Moreover, fisheries with known poor stock status may use pre-assessment as an endorsement to conduct formal stock assessments or as benchmark for amendment of management plans towards MSC certification [22].

### Comparing Rates of Poor Performance

Fisheries of most concern are those with biomass below *B*_MSY_ and continued high harvest rates, which hinder stock rebuilding to sustainable levels (*u*>*u*_MSY_; top left sector of Fig. 1A). Four certified stocks (9% of those analyzed) fell into this category, including North Sea saithe (*Pollachius virens*), North Sea sole (*Solea solea*), Atlantic Iberian sardine (*Sardina pilchardus*) and deep-water Cape hake (*Merluccius paradoxus*) (Table S1), compared with 51 (28%) uncertified stocks (Table S3) and 11 (44%) non-recommended stocks. In other words, certified fisheries are 3–5 times less likely to be subject to harmful overfishing than uncertified fisheries. At the time of the original MSC assessment, all three European stocks met certification requirements, as they were above the limit reference points defined by the International Council for the Exploration of the Seas (ICES). However, for the Iberian sardine, the mandatory annual audit revealed poor stock condition (i.e., below the limit reference point where recruitment to the population could be impaired) causing the suspension of the MSC certificate in January 2012, which will remain in place until the stock recovers. The other 3 stocks (North Sea saithe, North Sea sole and deep-water Cape hake) are currently well above their respective limit reference points as defined by the relevant advisory bodies [14], [28].

The MSC Standard requires stocks to be above a limit reference point, which itself is above the point at which recruitment is impaired, and requires that if this point is not empirically determined, a biomass level of 0.5*B*_MSY_ could be used as an acceptable proxy. The U.S. also uses 0.5*B*_MSY_ as a default Minimum Stock Size Threshold (MSST), below which stocks are classified as “overfished” [26]. In our analysis, 46 (27%) uncertified stocks had biomass levels below 0.5*B*_MSY_, compared with 4 (9%) certified stocks: North Sea saithe and Atlantic Iberian sardine as previously described, Eastern Baltic cod (*Gadus morhua*) and North Sea haddock (*Melanogrammus aeglefinus*). With the exception of the Atlantic Iberian sardine, which has had its MSC certificate removed, all 3 stocks are above the limit reference points defined by ICES without signs of recruitment overfishing [29]. Moreover, Eastern Baltic cod is under a strict rebuilding plan, which has resulted in an 80% reduction in exploitation rate and a three-fold increase in spawning biomass over the last five years [30] and North Sea haddock has experienced a reduction of the exploitation rate of 55% in the last 5 years [31].

As a target, *B*_MSY_ is often conditionally defined as the biomass that produces the maximum sustainable yield “under existing environmental conditions” [32]. Thus, even under an MSY control rule where *B*_MSY_ is the target stock biomass level, natural variation in productivity will result in stock fluctuations, being half of the time below *B*_MSY_ and half of the time above *B*_MSY_ [33]. Similarly, harvest rates might exceed *u*_MSY_ in some years. This natural population variability (typically driven by recruitment fluctuations in marine fishes) precludes the possibility of keeping stocks at constant levels. Successful management therefore must include continuous monitoring and an ability to adjust and enforce harvest levels [33], [34]. It is for these reasons that stocks can be certified, and retain their certification even when they drop below *B*_MSY_, provided they include proper management feedbacks and precautionary limits. Specifically, fisheries must have a management system in place that will detect decreases in biomass and respond by reducing the exploitation rate to a level that should enable recovery (i.e., harvest control rules). These feedback mechanisms present in certified fisheries result in well-managed fisheries fluctuating around their target reference points. Fisheries must meet these and other criteria in terms of ecosystem impacts and compliance with local and international laws to achieve and maintain certification [19].

### Weighting Results by Stock Size

By using individual fish stocks as our unit of analysis we weight all stocks equally. However, this does not account for the fact that their total biomass can differ by several orders of magnitude. Recent annual landings of certified stocks range from 7 metric tons for the North-eastern inshore sea bass (*Dicentrarchus labrax*) fishery to 1.7 million tons for the Pacific skipjack (*Katsuwonus pelamis*) fishery (Table S1). While 179 uncertified fisheries are considered in the present analysis, their combined landings are lower than the total landings of the 45 certified fisheries analyzed (6.8 vs. 8.0 million metric tons; Tables S1 and S2). When we compared the biomass and exploitation rates in relation to MSY reference points for the 10 largest certified and uncertified stocks we found that 8 of the certified stocks, including the largest which represent almost 6 million metric tons of landed seafood, are at or above *B*_MSY_, and are harvested at rates that should maintain the stocks above or fluctuating around their reference points (i.e., *u_current_*≤*u*_MSY;_ Table 1). Notably, EBS walleye pollock, which has been the target of many criticisms due to declines in biomass since certification [11], [15], [17], currently has a biomass level 25% higher than the target level (*B*_MSY_) and an exploitation rate that is less than half of *u*_MSY_ (Table 1). In contrast, most of the 10 largest uncertified stocks have biomass levels substantially lower than *B*_MSY_ and exploitation rates considerably higher than *u*_MSY_ (Table 1).

**Table 1:** Stock status by landings.

Stock name	Species	Large Marine Ecosystem	Landings (MT)	Most recent yearwith data	*B_current_/B_MSY_*	*u_current_/u_MSY_*
**Certified**						
Skipjack tuna	*Katsuwonus pelamis*	Pacific High Seas	1,700,000	2010	2.67	0.34
Herring	*Clupea harengus*	North East Atlantic	1,687,371	2010	1.24	1.05
Bering Sea walleye pollock	*Theragra chalcogramma*	East Bering Sea	813,000	2011	1.25	0.46
North East Atlantic mackerel*	*Scomber scombrus*	Celtic-Biscay Shelf	734,889	2010	1.37	1.27
Barents Sea Atlantic cod	*Gadus morhua*	Barents Sea	523,430	2010	1.02	0.58
Barents Sea saithe	*Pollachius virens*	Barents Sea	520,529	2010	1.08	0.99
Pacific hake	*Merluccius productus*	California Current	216,910	2010	1.75	0.82
Barents Sea haddock	*Melanogrammus aeglefinus*	Barents Sea	200,512	2010	1.20	0.71
North Sea herring	*Clupea harengus*	North Sea	168,443	2010	0.93	0.47
North Sea saithe	*Pollachius virens*	North Sea	161,462	2010	0.37	1.27
***Median***					***1.22***	***0.77***
**Uncertified**						
Chilean Jack Mackerel	*Trachurus murphyi*	Humboldt Current	744,495	2010	0.09	3.66
Blue Whiting Northeast Atlantic	*Micromesistius poutassou*	Iceland Shelf	634,978	2010	0.29	1.01
Yellowfin tuna Central Western Pacific	*Thunnus albacares*	Pacific High Seas	413,418	2005	1.29	0.80
Capelin Iceland	*Mallotus villosus*	Iceland Shelf	391,000	2010	0.40	0.01
Yellowfin tuna Indian Ocean	*Thunnus albacares*	Indian Ocean	325,854	2009	1.02	1.15
Capelin Barents Sea	*Mallotus villosus*	Barents Sea	323,000	2010	1.01	0.27
Sandeel North Sea Dogger Bank SA1	*Ammodytes marinus*	North Sea	285,794	2010	1.86	0.28
Yellowfin tuna Eastern Pacific	*Thunnus albacares*	Pacific High Seas	255,923	2010	0.71	1.13
Sardine South Africa	*Sardinops sagax*	Benguela Current	217,138	2006	0.75	0.55
Argentine hake Southern Argentina	*Merluccius hubbsi*	Patagonian Shelf	212,618	2008	0.40	1.49
***Median***					***0.73***	***0.91***

Biomass status and exploitation rate in relation to MSY reference points for the 10 largest certified and uncertified stocks by landings (metric tons, in 2010). Rows in ***bold italics*** represent median values for certified and uncertified stocks.

* MSC certificate currently suspended.

### Evaluating Performance against Conservation Targets

The sustainable yield in an ecosystem context depends on both the trophic level of the species [35] and the structure of the ecosystem [36]. As a result, the target biomass associated with an ecologically sustainable yield is unknown for each stock, and may be higher or lower than *B*_MSY_ depending on the species. To account for this, we also considered the more conservative target biomass of 1.3 *B*_MSY_ [37]. We found that 49% of the certified stocks were above 1.3 *B*_MSY_, compared with 29% of the uncertified fisheries and 4% of non-recommended fisheries (Table S3). However, such conservative target biomass reference points should be evaluated on a case by case basis taking into consideration biological, economic and social aspects of fisheries [38].

### Long-term Stock Performance

**Figure 2:**
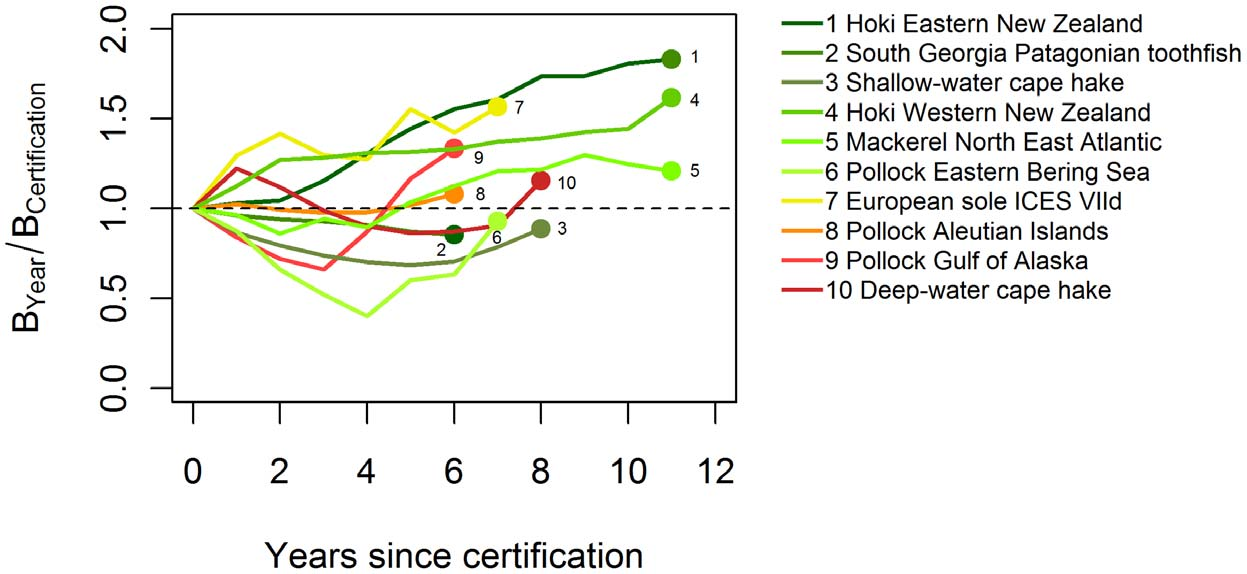
Time trends of current biomass (total or spawning) relative to biomass levels at the time of MSC assessment for stocks with available information and more than 5 years of certification. Colors represent current stock status (green to yellow: *B*>*B*MSY; orange to red: *B*<*B*MSY) and dots represent most recent year of available information (as per Table S1).

Given the relatively young age of the MSC, with 40% of stocks and 65% of fisheries certified in the last two years, trends in population biomass for individual fisheries after certification could only be analyzed for 10 certified stocks (23% of those analyzed) that had more than 5 years of available data after certification (Fig. 2; Table S1). For 7 out of 10 stocks, a combination of favorable environmental conditions, improved compliance to catch quotas (total allowable catch) as part of a rebuilding plan, and multiple management regulations (e.g., spatial and temporal closures, minimum landing sizes) have contributed to observed biomass increases and in some cases a rapid recovery in abundance to sustainable levels [30], [31] (Fig. 2). The rest of the analyzed stocks (3 out of 10) have shown slight declines in biomass since certification but remain above *B*_MSY_.

This fluctuation of stock size over time is evident in the time series data of biomass in relation to *B*_MSY_ after certification (Fig. 2). However, whether the changes over time seen in the 10 stocks with sufficient post-certification data are the product of good management depends on the initial biomass in relation to the target at the time the fishery was certified. South Georgia Patagonian toothfish (*Dissostichus eleginoides*), for example, declined in biomass since certification, but was still 22% larger than *B*_MSY_ in 2009. Conversely, New Zealand’s eastern and western hoki (*Macruronus novaezelandiae*) stocks were below *B*_MSY_ and certified under a rebuilding plan, and have since increased 300% in biomass. EBS walleye pollock and Northeast Atlantic mackerel (*Scomber scombrus*) both dropped below *B*_MSY_ after being certified but have improved in recent years and are now above *B*_MSY_. Certification of EBS walleye pollock was criticized [16] because the biomass fell 64% between 2004 and 2009. However, climate regime shifts in this region in the 1970s [39] greatly increased pollock productivity leading to a marked surge in biomass. For example, pollock spawning biomass in the 2000s has averaged 3.3 times that in the 1960s and is currently 5 times higher than when the fishery developed in 1964 [40]. Finally, for those stocks currently below *B*_MSY_, biomass has increased since the time of certification (Fig. 2). Although determining a causal connection between certification and increase in biomass would require an evaluation of reference uncertified fisheries and longer time series, the observed patterns are consistent with conservative harvest levels and responsive management systems required under MSC certification standards.

**Figure 3:**
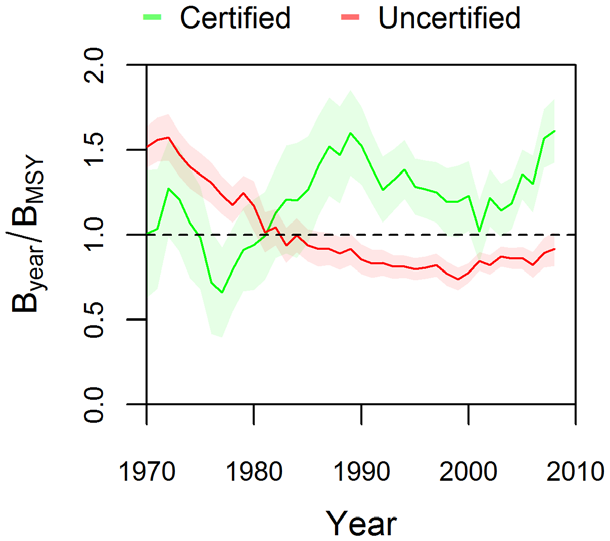
Performance of MSC-certified and uncertified fisheries. Long term trends (1970–2009) of biomass relative to their targets levels (i.e., estimated biomass at which the maximum sustainable yield should be obtained: *B*_MSY_; median ±S.E.). *B*_MSY_ is set to 1 (broken line).

Natural variability, changes in fisheries management systems, and adjustments in the MSC’s standards would likely affect the performance of certified fisheries against the standards in a particular year. When we examined four decades (1970-present) of data to characterize long-term trends in biomass relative to *B*_MSY_ we found that certified stocks on average performed better over the long-term than uncertified fisheries (Fig. 3). Biomass of uncertified fish stocks globally has been below *B*_MSY_ since the 1970s but shows signs of recovery towards *B*_MSY_ since 2000, while certified stocks have on average been consistently above *B*_MSY_ since 1980 (biomass long-term average  = 1.3*B*_MSY_). It is possible that differences early in the time series may reflect differences in how and when fisheries developed. However, MSC began certifying fisheries in 1999, while certified and uncertified stocks diverged from each other in the 1980s. This suggests that the stocks certified by MSC were performing well prior to certification. This improved performance has continued over the last 10 years, with certified stocks experiencing an average 45% increase in biomass compared with a 9% increase for uncertified stocks (Fig. 3; Table S5).

### Conclusions

Successful single-species management is only one part of fostering sustainable fisheries. There has been increasing support to move away from the single-species paradigm and towards an ecosystem-based approach [41], [42] which recognizes that fishing has both direct and indirect effects on marine systems [43]. Conserving ecosystem diversity and structure will play an important role in helping to retain ecosystem function in the face of climate change [44], [45]. While the impacts of fishing on ecosystems are difficult to measure, a few studies have attempted to quantify damage done not only by different types of gear but also by the volume of gear in the water [46], [47]. Through their certification standards the MSC has the opportunity to recognize fisheries that limit the collateral impacts of fishing on food webs, habitats, and ecosystems structure. However, more research is needed to quantify the performance of certified fisheries in these areas in comparison to uncertified fisheries [22].

Most certified fisheries come from developed countries with strong central governments, sophisticated fisheries management and data-rich situations. The analyzed time series (Fig. 3) suggests that these fisheries were already well managed by their agencies before certification. Given the increase in the number of fisheries seeking MSC certification in the last three years, future analyses will be able to examine the effect of MSC certification on initially less well managed fisheries, particularly small-scale and data-limited fisheries, which are critically important to developing world economies in terms of employment, national food security, and foreign exchange earnings [1]. An open question is whether certification and eco-label programs should raise the bar of sustainability, which may result in decreased market opportunities for small-scale and data-limited fisheries, or attempt to catalyze positive changes in those fisheries with informal and traditional management that are characteristic of many parts of the developing world, and in fisheries with current poor performance. These fisheries must be part of the solution in the pursuit of sustainable fisheries on a global scale.

Our study reveals that MSC-certified stocks are on average more likely to meet or exceed MSY-based target reference points, with higher biomass and lower exploitation rates than uncertified stocks. Certified stocks are also more likely to meet more conservative targets than uncertified stocks. Further, for those stocks with lower biomass levels, rebuilding plans are in place to improve stock health. While our time series analysis indicates that the observed difference in performance between certified and uncertified stocks existed prior to certification, the MSC eco-label is a reliable indicator of target stock health to consumers. It is important to note that MSC-certified fisheries not only must pass the sustainability criterion for the target stock but also must minimize ecosystem impact and have robust management systems. As more agencies attempt to implement ecosystem-based management, certified fisheries will need to demonstrate enhanced performance in these other areas to meet evolving definitions of sustainability and maintain the integrity of the MSC eco-label. A key part of MSC certification is the chain of custody to ensure that seafood labeled as MSC-certified indeed comes from the certified fishery and is not mislabeled catch from uncertified fisheries [48], [49]. Nevertheless, the current study shows that certification and eco-labeling can effectively recognize healthy stocks and fisheries that are achieving internationally accepted management targets. This is a critical first step in providing a mechanism for consumers to effectively influence change in fishing practices and ensure future ocean health and productivity.

## Supporting Information

Table S1 Summary information on certified stocks and their estimated current biomass and exploitation rates relative to MSY reference points (*B*_current_/*B*_MSY_ and *u*_current_/*u*_MSY_ or *F*_current_/*F*_MSY_). Rows in grey indicate certified stocks without available reference points or biomass estimates. These stocks were not used in the analysis (Fig. 1A). “Method used” indicates the method used to estimate *B*_MSY_ and *F*_MSY_ or *u*_MSY_: 1 =  stock assessment model, 2 =  surplus production model, and 3 = combination.
(DOCX)

Table S2 Summary information on uncertified stocks and their estimated current biomass and exploitation rates relative to MSY reference points (*B*/*B*_MSY_ and *u*/*u*_MSY_ or *F*/*F*_MSY_). “Method used” indicates the method used to estimate *B*_MSY_ and *u*_MSY_ or *F*_MSY_: 1 =  stock assessment model, 2 =  surplus production model, and 3 =  combination.
(DOCX)

Table S3 Median (±SE) biomass and exploitation rates relative to their targets and differences among certified, uncertified and not-recommended stocks. *denotes statistical significance (*P<0.05; **P<0.005).
(DOCX)

Table S4 Median biomass and exploitation rates relative to their targets and differences among certified, uncertified and not-recommended stocks for those fisheries with both *B*_MSY_ and *F*_MSY_ available from stock assessments
(DOCX)

Table S5 Results of test for difference in mean *B/B*_MSY_ over time between certified and uncertified stocks. *denotes significance (*P<0.05; **P<0.005).
(DOCX)

Text S1 This supporting information file includes expanded descriptions of methods used, additional results and model outputs and supporting references.
(DOCX)
