# Correction: Eco-Label Conveys Reliable Information on Fish Stock Health to Seafood Consumers

**DOI:** 10.1371/journal.pone.0210844

**Published:** 2019-01-10

**Authors:** Nicolás L. Gutiérrez, Sarah R. Valencia, Trevor A. Branch, David J. Agnew, Julia K. Baum, Patricia L. Bianchi, Jorge Cornejo-Donoso, Christopher Costello, Omar Defeo, Timothy E. Essington, Ray Hilborn, Daniel D. Hoggarth, Ashley E. Larsen, Chris Ninnes, Keith Sainsbury, Rebecca L. Selden, Seeta Sistla, Anthony D. M. Smith, Amanda Stern-Pirlot, Sarah J. Teck, James T. Thorson, Nicholas E. Williams, and the PLOS ONE Editors

There is additional information that needs to be included in the Competing Interests statement. The correct Competing Interests statement is as follows: NG, DJA, PLB, DDH, CN and ASP were employees of the Marine Stewardship Council at the time of this study. TEE, KS, and ADMS were unpaid members of the MSC's Technical Advisory Board. KS was an unpaid member of the MSC’s Board of Trustees. SRV, TAB, JKB, JCD, CC, OD, RH, AEL, RLS, SS, SJT, JTT and NEW did not have direct affiliation with MSC at the time of this study. The groups combined their initially separate but duplicative analyses as part of a National Center for Ecological Analysis and Synthesis (NCEAS) working group. KS additionally declares the following roles: Commissioner with the Australian Fishery Management Authority (since 2004), member of the Lenfest Task Force to develop standards for management of fisheries targeted on forage fish species (2008–2012), independent advisor to the Corporate Social Responsibility Committee of the China Fisheries Group (2011–2015), and consultant to the Food and Agriculture Organization of the United Nations, authoring a 2008 review of fisheries ecolabelling schemes. Funding was received by RH in the five-year period prior to the publication of this article from sources including fishery/seafood corporations and industry groups, as well as environmental groups and foundations (please see the list attached here as Supporting Information S1 File). RH has previously received income from Tavel Certification Inc. and Scientific Certification Systems, which are certification companies approved by the Marine Stewardship Council; the activities of the Marine Stewardship Council are the subject of the current article. While this funding did not directly support the research reported in this article, the authors are declaring it in accordance with *PLOS ONE* policy. This does not alter the authors' adherence to *PLOS ONE* policies on sharing data and materials.

The status of six co-authors as employees of the Marine Stewardship Council was declared by the authors at the time of submission but was removed from the Competing Interests section in an error by the journal office. The publisher apologizes for this error. The funding that directly supported the work reported in this article is already listed accurately and in full in the Funding statement.

## Supporting information

S1 File Funding received by RH during the 5 years prior to publication of this article. (XLSX)
