# Supplementary material for: Eco-Label Conveys Reliable Information on Fish Stock Health to Seafood Consumers
Source: PLoS One. 2012 Aug 21;7(8):e43765. doi: 10.1371/journal.pone.0043765 (PMC3424161; doi:10.1371/journal.pone.0043765)
Supplement: Table S1 — Summary information on certified stocks and their estimated current biomass and exploitation rates relative to MSY reference points (B current/B MSY and u current/u MSY or F current/F MSY). Rows in grey indicate certified stocks without available reference points or biomass estimates. These stocks were not used in the analysis (Fig. 1A). “Method used” indicates the method used to estimate B MSY and F MSY or u MSY: 1 = stock assessment model, 2 = surplus production model, and 3 = combination. (DOCX) [file pone.0043765.s001.docx]

**Table S1.** Summary information on certified stocks and their estimated current biomass and exploitation rates relative to MSY reference points (*B*_current_/*B*_MSY_ and *u*_current_/*u*_MSY_ or *F*_current_/*F*_MSY_). Rows in grey indicate certified stocks without available reference points or biomass estimates. These stocks were not used in the analysis (Fig. 1A). “Method used” indicates the method used to estimate *B*_MSY_ and *F*_MSY_ or *u*_MSY_: 1 = stock assessment model, 2 = surplus production model, and 3=combination.

| **Large Marine Ecosystem** | **Scientific name** | **Stock and common name** | **Year of assessment** | ***B*/*B*_MSY_** | ***F*/*F*_MSY_** | **Method used** | **Reference** |
| --- | --- | --- | --- | --- | --- | --- | --- |
| Atlantic High Seas | *Xiphias gladius* | North West Atlantic swordfish | |  |  |  |  |
| Atlantic High Seas | *Xiphias gladius* | Swordfish | 2008 | 1.05 | 0.77 | 1 | S5 |
| Baltic Sea | *Gadus morhua* | Eastern Baltic cod | 2010 | 0.31 | 0.83 | 3 | S6 |
| Barents Sea | *Pollachius virens* | Barents Sea saithe | 2010 | 1.08 | 0.99 | 1 | S7 |
| Barents Sea | *Gadus morhua* | Atlantic cod | 2010 | 1.02 | 0.58 | 1 | S7 |
| Barents Sea | *Melanogrammus aeglefinus* | Barents Sea haddock | 2010 | 1.20 | 0.71 | 3 | S7 |
| Benguela Current | *Merluccius capensis* | Shallow-water cape hake | 2011 | 1.81 | 0.21 | 1 | S8 |
| Benguela Current | *Merluccius paradoxus* | Deep-water cape hake | 2011 | 0.88 | 1.07 | 1 | S8 |
| California Current | *Panulirus interuptus* | Baja California red rock lobster | 2010 | 1.50 | 0.50 | 1 | S9 |
| California Current | *Hippoglossus stenolepis* | Pacific halibut | 2010 | 1.43 | 1.22 | 1 | S10 |
| California Current | *Pandalus jordani* | Oregon pink shrimp |  |  |  |  |  |
| California Current | *Merluccius productus* | Pacific hake | 2010 | 1.75 | 0.82 | 1 | S11 |
| California Current | *Anoplopoma fimbria* | Sablefish | 2011 | 0.76 | 0.85 | 1 | S12 |
| California Current | *Cancer magister* | Oregon Dungeness crab |  |  |  |  |  |
| Celtic-Biscay Shelf | *Scomber scombrus* | North East Atlantic mackerel | 2010 | 1.37 | 1.27 | 3 | S13 |
| Celtic-Biscay Shelf | *Solea solea* | Eastern Channel sole (VIId) | 2010 | 1.35 | 1.56 | 3 | S14 |
| Celtic-Biscay Shelf | *Nephrops norvegicus* | Nephrops North | 2010 | 1.49 | 0.61 | 3 | S15 |
| Celtic-Biscay Shelf | *Nephrops norvegicus* | Nephrops South | 2010 | 1.86 | 0.60 | 3 | S15 |
| Celtic-Biscay Shelf | *Sardina pilchardus* | Iberian sardine | 2010 | 0.32 | 1.37 | 2 | S16 |
| East Bering Sea | *Pleuronectes q.* | Alaska plaice | 2010 | 1.47 | 0.03 | 1 | S17 |
| East Bering Sea | *Lepidopsetta polyxystra* | Northern rock sole | 2010 | 2.06 | 0.61 | 1 | S18 |
| East Bering Sea | *Limanda aspera* | Yellowfin sole | 2010 | 1.75 | 0.57 | 1 | S19 |
| East Bering Sea | *Oncorhynchus gorbuscha* | Alaska salmon |  |  |  |  |  |
| East Bering Sea | *Oncorhynchus keta* | Alaska salmon |  |  |  |  |  |
| East Bering Sea | *Oncorhynchus kisutch* | Alaska salmon |  |  |  |  |  |
| East Bering Sea | *Oncorhynchus nerka* | Alaska salmon |  |  |  |  |  |
| East Bering Sea | *Oncorhynchus tshawytscha* | Alaska salmon |  |  |  |  |  |
| East Bering Sea | *Theragra chalcogramma* | Aleutian Is. walleye pollock | 2010 | 0.74 | 0.03 | 1 | S20 |
| East Bering Sea | *Theragra chalcogramma* | Bering Sea walleye pollock | 2010 | 1.25 | 0.37 | 1 | S21 |
| East Bering Sea | *Anoplopoma fimbria* | Sablefish | 2010 | 0.93 | 0.68 | 1 | S22 |
| East Bering Sea | *Reinhardtius stomias* | Arrowtooth flounder | 2010 | 2.68 | 0.10 | 1 | S23 |
| East Bering Sea | *Hippoglossoides elassodon* | Flathead sole | 2010 | 1.75 | 0.26 | 1 | S24 |
| East Bering Sea | *Gadus macrocephalus* | Pacific cod | 2010 | 0.86 | 0.88 | 1 | S25 |
| Gulf of Alaska | *Theragra chalcogramma* | Walleye pollock | 2010 | 0.78 | 0.42 | 1 | S26 |
| Gulf of Alaska | *Gadus macrocephalus* | Pacific cod | 2010 | 1.21 | 0.71 | 1 | S27 |
| Gulf of Alaska | *Reinhardtius stomias* | Arrowtooth flounder | 2010 | 2.62 | 0.09 | 1 | S28 |
| Gulf of Alaska | *Hippoglossoides elassodon* | Flathead sole | 2010 | 2.40 | 0.02 | 1 | S29 |
| Gulf of Alaska | *Glyptocephalus zachirus* | Rex sole | 2009 | 2.30 | 0.28 | 1 | S30 |
| Kuroshio Current | *Hippoglossoides dubius* | Kyoto flathead flounder |  |  |  |  |  |
| Kuroshio Current | *Chionoecetes opilio* | Kyotosnow crab |  |  |  |  |  |
| Kuroshio Current | *Oncorhynchus gorbuscha* | Iturup Island pink salmon |  |  |  |  |  |
| Kuroshio Current | *Oncorhynchus keta* | Iturup Island chum salmon |  |  |  |  |  |
| N/A | *Sander lucioperca* | Lake Hjälmaren pikeperch |  |  |  |  |  |
| New Zealand Shelf | *Macruronus novaezelandiae* | Eastern New Zealand hoki | 2011 | 2.29 | 0.45 | 1 | S31 |
| New Zealand Shelf | *Macruronus novaezelandiae* | Western New Zealand hoki | 2011 | 1.80 | 0.71 | 1 | S31 |
| North East Atlantic | *Clupea harengus* | North East Atlantic herring | 2010 | 1.24 | 1.05 | 3 | S12 |
| North Sea | *Solea solea* | North Sea sole | 2010 | 0.73 | 1.54 | 3 | S32 |
| North Sea | *Clupea harengus* | North Sea herring | 2009 | 0.93 | 0.47 | 3 | S33 |
| North Sea | *Dicentrarchus labrax* | North Eastern Sea sea bass |  |  |  |  |  |
| North Sea | *Pollachius virens* | North Sea saithe | 2010 | 0.37 | 1.27 | 3 | S34 |
| North Sea | *Mytilus edulis* | Denmark blue shell mussel |  |  |  |  |  |
| North Sea | *Mytilus edulis* | North Menai Strait mussel |  |  |  |  |  |
| North Sea | *Melanogrammus aeglefinus* | North Sea haddock | 2010 | 0.41 | 0.77 | 3 | S34 |
| North Sea | *Pleuronectes platessa* | European Plaice | 2010 | 1.04 | 0.96 | 3 | S34 |
| Northeast U.S. Shelf | *Limanda ferruginea* | Yellowtail Flounder | 2010 | 1.64 | 0.15 | 1 | S35 |
| Northeast U.S. Shelf | *Homarus americanus* | Eastern Canada offshore lobster | |  |  |  |  |
| Northeast U.S. Shelf | *Geryon quinquedens* | Atlantic deep sea red crab |  |  |  |  |  |
| Northeast U.S. Shelf | *Melanogrammus aeglefinus* | Haddock 4X5Y | 2009 | 1.14 | 0.37 | 1 | S34 |
| Northeast U.S. Shelf | *Melanogrammus aeglefinus* | Haddock 5Zejm | 2009 | 1.13 | 0.67 | 1 | S37 |
| Pacific High Seas | *Thunnus alalunga* | South Pacific albacore tuna | 2006 | 2.46 | 0.91 | 1 | S38 |
| Pacific High Seas | *Katsuwonus pelamis* | Skipjack tuna | 2010 | 2.67 | 0.34 | 1 | S39 |
| Pacific High Seas | *Thunnus alalunga* | North Pacific albacore tuna |  |  |  |  |  |
| Patagonian shelf | *Zygochlamys patagonica* | Patagonian scallop |  |  |  |  |  |
| Scotian Shelf | *Pandalus borealis* | Gulf of St. Lawrence shrimp |  |  |  |  |  |
| Scotian Shelf | *Placopecten magellanicus* | Eastern Canada offshore scallop | |  |  |  |  |
| South China Sea | *Meretrix lyrata* | Vietnam Ben Tre clam |  |  |  |  |  |
| Australian Shelf | *Donax deltoides* | Lakes and Coorong cockle |  |  |  |  |  |
| Australian Shelf | *Aldrichetta forsteri* | Lakes and Coorong mullet |  |  |  |  |  |
| Australian Shelf | *Argyrosomus hololepidotus* | Lakes and Coorong mulloway | |  |  |  |  |
| Australian Shelf | *Macquaria ambigua* | Lakes and Coorong perch |  |  |  |  |  |
| Australian Shelf | *Panulirus cygnus* | Western Australia rock lobster | |  |  |  |  |
| Subantarctic High Seas | *Dissostichus eleginoides* | South Georgia toothfish | 2010 | 1.05 | 0.90 | 1 | S40 |
| Subantarctic High Seas | *Champsocephalus gunnari* | South Georgia icefish |  |  |  |  |  |
| Subantarctic High Seas | *Euphausia superba* | Antarctic krill |  |  |  |  |  |
| Subantarctic High Seas | *Dissostichus mawsoni* | Ross Sea toothfish | 2010 | 1.60 | 1.00 | 1 | S41 |
|  |  |  |  |  |  |  |  |
