# Supplementary material for: Eco-Label Conveys Reliable Information on Fish Stock Health to Seafood Consumers
Source: PLoS One. 2012 Aug 21;7(8):e43765. doi: 10.1371/journal.pone.0043765 (PMC3424161; doi:10.1371/journal.pone.0043765)
Supplement: Table S2 — Summary information on uncertified stocks and their estimated current biomass and exploitation rates relative to MSY reference points (B/B MSY and u/u MSY or F/F MSY). “Method used” indicates the method used to estimate B MSY and u MSY or F MSY: 1 = stock assessment model, 2 = surplus production model, and 3 = combination. (DOCX) [file pone.0043765.s002.docx]

**Table S2.** Summary information on uncertified stocks and their estimated current biomass and exploitation rates relative to MSY reference points (*B*/*B*_MSY_ and *u*/*u*_MSY_ or *F*/*F*_MSY_). “Method used” indicates the method used to estimate *B*_MSY_ and *u*_MSY_ or *F*_MSY_: 1 = stock assessment model, 2 = surplus production model, and 3 = combination.

| **Stock name** | **Species** | **Large Marine Ecosystem** | **Assessment Agency** | **Recent Landings (MT)** | **Current year** | ***B/B_MSY_*** | ***F/F_MSY_*** | **Method used** | **Source** |
| --- | --- | --- | --- | --- | --- | --- | --- | --- | --- |
| Albacore tuna North Atlantic | *Thunnus alalunga* | Atlantic High Seas | ICCAT | 36,198.4 | 2005 | 0.81 | 1.49 | 2 | S2 |
| American lobster Rhode Island | *Homarus americanus* | Northeast U.S. Shelf | RIDEM | 655.2 | 2006 | 0.53 | 0.67 | 1 | S2 |
| American Plaice NAFO 3LNO | *Hippoglossoides platessoides* | Newfoundland-Labrador Shelf | NAFO | 2,828.0 | 2006 | 0.08 | 0.77 | 2 | S2 |
| American Plaice NAFO 5YZ | *Hippoglossoides platessoides* | Northeast U.S. Shelf | NEFSC | 1,226.2 | 2007 | 0.70 | 0.30 | 1 | S2 |
| Anchovy South Africa | *Engraulis encrasicolus* | Benguela Current | MARAM | 134,904.0 | 2006 | 0.97 | 0.36 | 2 | S2 |
| Argentine anchoita Northern Argentina | *Engraulis anchoita* | Patagonian Shelf | INIDEP | 24,494.1 | 2007 | 1.37 | 0.17 | 1 | S2 |
| Argentine anchoita Southern Argentina | *Engraulis anchoita* | Patagonian Shelf | INIDEP | 3,271.0 | 2007 | 3.13 | 0.04 | 1 | S2 |
| Argentine hake Northern Argentina | *Merluccius hubbsi* | Patagonian Shelf | INIDEP | 50,816.0 | 2007 | 0.19 | 1.26 | 1 | S2 |
| Argentine hake Southern Argentina | *Merluccius hubbsi* | Patagonian Shelf | INIDEP | 212,638.0 | 2008 | 0.40 | 1.49 | 1 | S2 |
| Arrowtooth flounder Pacific Coast | *Reinhardtius stomias* | California Current | NWFSC | 2,381.0 | 2007 | 3.81 | 0.21 | 1 | S2 |
| Atka mackerel BSAI | *P. monopterygius* | East Bering Sea | AFSC | 58,471.0 | 2009 | 1.55 | 0.55 | 1 | S2 |
| Atlantic cod Baltic Areas 22 and 24 | *Gadus morhua* | Baltic Sea | ICES | 15,290.0 | 2010 | 0.28 | 2.32 | 3 | S6 |
| Atlantic Cod Celtic Sea VIIe-k | *Gadus morhua* | Celtic-Biscay Shelf | ICES | 3,236.0 | 2010 | 0.34 | 1.28 | 3 | S14 |
| Atlantic cod coastal Norway | *Gadus morhua* | Norwegian Sea | ICES | 26,134.0 | 2010 | 0.25 | 1.88 | 2 | S7 |
| Atlantic cod Faroe Plateau | *Gadus morhua* | Faroe Plateau | ICES | 14,048.0 | 2010 | 0.60 | 1.30 | 3 | S40 |
| Atlantic cod Georges Bank | *Gadus morhua* | Northeast U.S. Shelf | NEFSC | 5,957.0 | 2007 | 0.12 | 0.72 | 1 | S2 |
| Atlantic cod Gulf of Maine | *Gadus morhua* | Northeast U.S. Shelf | NEFSC | 3,989.0 | 2007 | 0.63 | 2.40 | 1 | S2 |
| Atlantic cod Iceland | *Gadus morhua* | Iceland Shelf | ICES | 181,151.0 | 2009 | 0.52 | 0.95 | 2 | S41 |
| Atlantic cod Irish Sea | *Gadus morhua* | Celtic-Biscay Shelf | ICES | 468.0 | 2010 | 0.17 | 2.98 | 3 | S14 |
| Atlantic cod Kattegat | *Gadus morhua* | North Sea | ICES | 876.0 | 2010 | 0.04 | 2.02 | 2 | S2 |
| Atlantic cod NAFO 3NO | *Gadus morhua* | Newfoundland-Labrador Shelf | NAFO | 601.0 | 2006 | 0.02 | 0.27 | 2 | S2 |
| Atlantic cod NAFO 3Pn4RS | *Gadus morhua* | Newfoundland-Labrador Shelf | NAFO | 5,459.0 | 2006 | 0.09 | 0.79 | 2 | S2 |
| Atlantic cod North Sea | *Gadus morhua* | North Sea | ICES | 91,428.0 | 2010 | 0.21 | 3.53 | 3 | S33 |
| Atlantic cod West of Scotland | *Gadus morhua* | Celtic-Biscay Shelf | ICES | 4,505.2 | 2010 | 0.16 | 4.34 | 3 | S14 |
| Atlantic Halibut NAFO 5YZ | *Hippoglossus hippoglossus* | Northeast U.S. Shelf | NAFO | 84.2 | 2007 | 0.03 | 0.89 | 1 | S2 |
| Atlantic Herring Nothwestern Atlantic | *Clupea harengus* | Northeast U.S. Shelf | NEFSC | 105,897.6 | 2005 | 1.66 | 0.34 | 1 | S2 |
| Atlantic menhaden Atlantic | *Brevoortia tyrannus* | Northeast U.S. Shelf | ASMFC | 185,100.0 | 2005 | 0.47 | 0.97 | 2 | S2 |
| Australian salmon New Zealand | *Arripis trutta* | New Zealand Shelf | NZMF | 2,240.0 | 2006 | 1.64 | 0.33 | 1 | S2 |
| Bigeye tuna Atlantic | *Thunnus obesus* | Atlantic High Seas | ICCAT | 71,020.0 | 2009 | 1.05 | 0.95 | 1 | S42 |
| Bigeye tuna Eastern Pacific Ocean | *Thunnus obesus* | Pacific High Seas | SPC | 81,391.0 | 2010 | 1.24 | 1.13 | 1 | S43 |
| Bigeye tuna Western Pacific Ocean | *Thunnus obesus* | Pacific High Seas | SPC | 107,413.3 | 2006 | 1.06 | 1.38 | 2 | S44 |
| Black oreo West end of Chatham Rise | *Allocyttus niger* | New Zealand Shelf | NZMF | 1,766.7 | 2007 | 0.99 | 0.82 | 1 | S2 |
| Black rockfish Northern Pacific Coast | *Sebastes melanops* | California Current | NWFSC | 323.3 | 2006 | 1.77 | 0.47 | 1 | S2 |
| Black rockfish Southern Pacific Coast | *Sebastes melanops* | California Current | NWFSC | 627.2 | 2007 | 2.23 | 0.33 | 1 | S2 |
| Black sea bass Mid-Atlantic Coast | *Centropristis striata* | Northeast U.S. Shelf | NEFSC | 2,811.0 | 2007 | 1.00 | 0.67 | 1 | S2 |
| Blue rockfish California | *Sebastes mystinus* | California Current | NWFSC | 263.1 | 2007 | 0.75 | 1.19 | 1 | S2 |
| Blue Warehou Eastern SE Australia | *Seriolella brama* | Southeast Australian Shelf | CSIRO | 171.5 | 2006 | 0.49 | 0.84 | 1 | S2 |
| Blue Warehou Western SE Australia | *Seriolella brama* | Southwest Australian Shelf | CSIRO | 600.0 | 2006 | 0.41 | 2.04 | 1 | S2 |
| Blue Whiting Northeast Atlantic | *Micromesistius poutassou* | Iceland Shelf | ICES | 634,978.0 | 2010 | 0.29 | 1.01 | 3 | S2 |
| Bluefin tuna Eastern Atlantic | *Thunnus thynnus* | Atlantic High Seas | ICCAT | 60,714.7 | 2009 | 0.45 | 0.78 | 2 | S45 |
| Bluefin tuna Western Atlantic | *Thunnus thynnus* | Atlantic High Seas | ICCAT | 1,624.0 | 2007 | 0.57 | 1.33 | 2 | S46 |
| Bluefish Atlantic Coast | *Pomatomus saltatrix* | Northeast U.S. Shelf | NEFSC | 15,568.0 | 2007 | 0.81 | 0.79 | 1 | S2 |
| Bocaccio Southern Pacific Coast | *Sebastes paucispinis* | California Current | NWFSC | 67.0 | 2006 | 0.32 | 0.10 | 2 | S2 |
| Cabezon Northern California | *Scorpaenichthys marmoratus* | California Current | AFSC | 72.9 | 2005 | 1.04 | 0.99 | 2 | S2 |
| Cabezon Southern California | *Scorpaenichthys marmoratus* | California Current | AFSC | 12.8 | 2005 | 0.74 | 0.53 | 2 | S2 |
| Canary rockfish Pacific Coast | *Sebastes pinniger* | California Current | NWFSC | 40.5 | 2009 | 0.62 | 0.03 | 1 | S2 |
| Cape horse mackerel South Africa | *Trachurus capensis* | Agulhas Current | MARAM | 49,000.0 | 2007 | 1.47 | 0.76 | 2 | S2 |
| Capelin Barents Sea | *Mallotus villosus* | Barents Sea | ICES | 323,000.0 | 2010 | 1.01 | 0.27 | 2 | S7 |
| Capelin Iceland | *Mallotus villosus* | Iceland Shelf | ICES | 391,000.0 | 2010 | 0.40 | 0.01 | 2 | S2 |
| Chilean Jack Mackerel | *Trachurus murphyi* | Humboldt Current |  | 744,495.0 | 2010 | 0.09 | 3.66 | 2 | S2 |
| Chilipepper Southern Pacific Coast | *Sebastes goodei* | California Current | NWFSC | 127.0 | 2006 | 2.77 | 0.04 | 1 | S2 |
| Common European sole Bay of Biscay | *Solea vulgaris* | Celtic-Biscay Shelf | ICES | 3,600.0 | 2010 | 0.32 | 1.50 | 3 | S13 |
| Common European sole Celtic Sea | *Solea vulgaris* | Celtic-Biscay Shelf | ICES | 790.0 | 2009 | 1.24 | 0.84 | 3 | S47 |
| Common European sole Irish Sea | *Solea vulgaris* | Celtic-Biscay Shelf | ICES | 324.0 | 2010 | 0.19 | 1.68 | 3 | S14 |
| Common European sole Skaggerak | *Solea vulgaris* | North Sea | ICES | 640.0 | 2010 | 0.76 | 0.89 | 3 | S6 |
| Common gemfish New Zealand | *Rexea solandri* | New Zealand Shelf | NZMF | 412.0 | 2006 | 1.64 | 0.27 | 1 | S2 |
| Common gemfish SE Australia | *Rexea solandri* | Southeast Australian Shelf | CSIRO | 102.0 | 2007 | 0.25 | 0.39 | 2 | S2 |
| Cowcod Southern California | *Sebastes levis* | California Current | NWFSC | 0.5 | 2007 | 0.09 | 0.07 | 1 | S2 |
| Darkblotched rockfish Pacific Coast | *Sebastes crameri* | California Current | NWFSC | 113.0 | 2007 | 0.73 | 0.31 | 2 | S2 |
| Deepwater flathead SE Australia | *Platycephalus conatus* | Southwest Australian Shelf | CSIRO | 1,039.7 | 2007 | 1.51 | 0.61 | 2 | S2 |
| Dover sole Pacific Coast | *Microstomus pacificus* | California Current | SWFSC | 7,145.3 | 2005 | 1.61 | 0.45 | 1 | S2 |
| Dusky rockfish Gulf of Alaska | *Sebastes variabilis* | Gulf of Alaska | AFSC | 3,318.0 | 2007 | 1.54 | 0.54 | 1 | S2 |
| English sole Pacific Coast | *Parophrys vetulus* | California Current | NWFSC | 1,078.0 | 2007 | 6.42 | 0.14 | 1 | S2 |
| European Plaice Eastern Channel | *Pleuronectes platessa* | Celtic-Biscay Shelf | ICES | 3,800.0 | 2010 | 0.61 | 8.68 | 2 | S33 |
| European Plaice Irish Sea VIIa | *Pleuronectes platessa* | Celtic-Biscay Shelf | ICES | 456.0 | 2008 | 0.86 | 0.16 | 3 | S48 |
| European Plaice Western Channel | *Pleuronectes platessa* | Celtic-Biscay Shelf | ICES | 1,043.0 | 2010 | 0.99 | 2.38 | 3 | S14 |
| Gag Southern Atlantic Coast | *Mycteropercas microlepis* | Southeast U.S. Shelf | SEFSC | 320.7 | 2005 | 0.94 | 1.31 | 1 | S2 |
| Greenland halibut NAFO 23 | *Reinhardtius hippoglossoides* | Newfoundland-Labrador Shelf | NAFO | 24,000.0 | 2006 | 0.39 | 1.73 | 2 | S2 |
| Greenland turbot BSAI | *Reinhardtius hippoglossoides* | East Bering Sea | AFSC | 2,541.0 | 2009 | 1.48 | 0.05 | 1 | S2 |
| Haddock Faroe Plateau | *Melanogrammus aeglefinus* | Faroe Plateau | ICES | 5,183.0 | 2010 | 0.30 | 1.02 | 2 | S40 |
| Haddock Iceland | *Melanogrammus aeglefinus* | Iceland Shelf | ICES | 82,045.0 | 2010 | 0.58 | 1.36 | 2 | S40 |
| Haddock NAFO 5Y | *Melanogrammus aeglefinus* | Northeast U.S. Shelf | DFO | 1,368.0 | 2007 | 0.99 | 1.21 | 1 | S2 |
| Haddock Rockall VIb | *Melanogrammus aeglefinus* | Celtic-Biscay Shelf | ICES | 3,400.0 | 2010 | 0.52 | 2.13 | 3 | S14 |
| Haddock West of Scotland | *Melanogrammus aeglefinus* | Celtic-Biscay Shelf | ICES | 4,487.0 | 2009 | 0.22 | 0.96 | 3 | S47 |
| Hake Northeast Atlantic North | *Merluccius merluccius* | Celtic-Biscay Shelf | ICES | 41,800.0 | 2010 | 1.57 | 1.15 | 3 | S13 |
| Herring Gulf of Riga | *Clupea harengus* | Baltic Sea | ICES | 33,376.0 | 2010 | 1.08 | 1.23 | 3 | S6 |
| Herring ICES 25-32 | *Clupea harengus* | Baltic Sea | ICES | 132,135.0 | 2010 | 0.39 | 1.98 | 3 | S6 |
| Herring ICES 30 | *Clupea harengus* | Baltic Sea | ICES | 68,873.0 | 2010 | 1.31 | 0.70 | 3 | S6 |
| Herring ICES 31 | *Clupea harengus* | Baltic Sea | ICES | 2,954.0 | 2010 | 2.11 | 0.12 | 2 | S6 |
| Herring ICES VIa | *Clupea harengus* | Celtic-Biscay Shelf | ICES | 18,508.0 | 2010 | 0.82 | 1.06 | 3 | S32 |
| Herring Western Baltic Spring | *Clupea harengus* | Baltic Sea | ICES | 42,000.0 | 2010 | 0.31 | 1.20 | 3 | S32 |
| Herring Northern Irish Sea | *Clupea harengus* | Celtic-Biscay Shelf | ICES | 4,402.0 | 2010 | 0.26 | 1.04 | 2 | S32 |
| Herring Southern Irish Sea | *Clupea harengus* | Celtic-Biscay Shelf | ICES | 8,370,000 | 2010 | 0.82 | 0.34 | 3 | S32 |
| Horse Mackerel Western Stock | *Trachurus trachurus* | Iceland Shelf | ICES | 204,000.0 | 2010 | 1.04 | 1.02 | 3 | S12 |
| Jackass morwong Southeast Australia | *Nemadactylus macropterus* | Southeast Australian Shelf | CSIRO | 396.0 | 2007 | 0.31 | 1.80 | 2 | S2 |
| Kingklip South Africa | *Genypterus capensis* | Benguela Current | MARAM | 2,404.0 | 2008 | 1.20 | 0.55 | 2 | S2 |
| Longnose skate Pacific Coast | *Raja rhina* | California Current | NWFSC | 899.2 | 2007 | 1.76 | 0.40 | 1 | S2 |
| Longspine thornyhead Pacific Coast | *Sebastolobus altivelis* | California Current | NWFSC | 912.0 | 2005 | 2.65 | 0.23 | 1 | S2 |
| Monkfish Gulf of Maine | *Lophius americanus* | Northeast U.S. Shelf | NEFSC | 7,187.0 | 2006 | 2.42 | 1.23 | 1 | S2 |
| Monkfish Southern Georges Bank | *Lophius americanus* | Northeast U.S. Shelf | NEFSC | 9,561.0 | 2006 | 1.72 | 0.30 | 2 | S2 |
| New Zealand abalone PAU 5A | *Haliotis iris* | New Zealand Shelf | NZMF | 156.7 | 2006 | 0.72 | 2.83 | 2 | S2 |
| New Zealand abalone PAU 5B | *Haliotis iris* | New Zealand Shelf | NZMF | 111.0 | 2007 | 1.02 | 0.59 | 2 | S2 |
| New Zealand abalone PAU 5D | *Haliotis iris* | New Zealand Shelf | NZMF | 110.8 | 2006 | 0.44 | 2.10 | 2 | S2 |
| New Zealand abalone PAU 7 | *Haliotis iris* | New Zealand Shelf | NZMF | 202.1 | 2008 | 0.87 | 0.94 | 2 | S2 |
| New Zealand ling E Australia | *Genypterus blacodes* | Southeast Australian Shelf | NZMF | 736.0 | 2007 | 0.59 | 2.20 | 2 | S2 |
| New Zealand ling LIN 6b | *Genypterus blacodes* | New Zealand Shelf | NZMF | 400.0 | 2006 | 2.19 | 0.11 | 1 | S2 |
| New Zealand ling LIN 72 | *Genypterus blacodes* | New Zealand Shelf | NZMF | 450.0 | 2007 | 2.49 | 0.32 | 1 | S2 |
| New Zealand ling LIN 7WC | *Genypterus blacodes* | New Zealand Shelf | NZMF | 2,400.0 | 2008 | 2.21 | 0.13 | 1 | S2 |
| New Zealand ling LIN 3 and 4 | *Genypterus blacodes* | New Zealand Shelf | NZMF | 4,100.0 | 2007 | 3.07 | 0.09 | 1 | S2 |
| New Zealand ling LIN 5 and 6 | *Genypterus blacodes* | New Zealand Shelf | NZMF | 8,000.0 | 2007 | 3.96 | 0.10 | 1 | S2 |
| New Zealand snapper Area 8 | *Chrysophrys auratus* | New Zealand Shelf | NZMF | 1,950.0 | 2005 | 0.35 | 2.50 | 1 | S2 |
| Northern rockfish BSAI | *Sebastes polyspinis* | East Bering Sea | AFSC | 885.0 | 2009 | 1.42 | 0.13 | 2 | S2 |
| Northern rockfish Gulf of Alaska | *Sebastes polyspinis* | Gulf of Alaska | AFSC | 4,187.0 | 2008 | 1.50 | 0.66 | 1 | S2 |
| Northern shrimp Gulf of Maine | *Pandalus borealis* | Northeast U.S. Shelf | ASMFC | 4,783.5 | 2008 | 1.58 | 0.56 | 2 | S2 |
| Norway pout North Sea | *Trisopterus esmarkii* | North Sea | ICES | 54.5 | 2010 | 2.16 | 0.06 | 2 | S33 |
| Orange roughy Southeast Australia | *Hoplostethus atlanticus* | Southeast Australian Shelf | CSIRO | 772.0 | 2007 | 0.52 | 0.29 | 1 | S2 |
| Pacific herring Central Coast | *Clupea pallasii* | Gulf of Alaska | DFO | 398.0 | 2007 | 0.30 | 0.11 | 2 | S2 |
| Pacific herring Prince Rupert District | *Clupea pallasii* | Gulf of Alaska | DFO | 968.0 | 2007 | 0.16 | 0.32 | 2 | S2 |
| Pacific herring Queen Charlotte Islands | *Clupea pallasii* | Gulf of Alaska | DFO | 0.0 | 2007 | 0.20 | 0.00 | 2 | S2 |
| Pacific herring Straight of Georgia | *Clupea pallasii* | Gulf of Alaska | DFO | 9,822.0 | 2007 | 0.91 | 0.40 | 2 | S2 |
| Pacific herring Vancouver Island | *Clupea pallasii* | Gulf of Alaska | DFO | 0.0 | 2007 | 0.03 | 0.00 | 2 | S2 |
| Pacific ocean perch BSAI | *Sebastes alutus* | East Bering Sea | AFSC | 12,627.0 | 2009 | 1.27 | 0.26 | 2 | S2 |
| Pacific ocean perch Gulf of Alaska | *Sebastes alutus* | Gulf of Alaska | AFSC | 12,953.0 | 2007 | 1.17 | 0.73 | 1 | S2 |
| Pacific ocean perch Pacific Coast | *Sebastes alutus* | California Current | NWFSC | 83.0 | 2007 | 0.69 | 0.00 | 1 | S2 |
| Pacific Sardine | *Sardinops sagax* | California Current | SWFSC | 149,789.0 | 2006 | 1.36 | 0.41 | 2 | S2 |
| Patagonian grenadier S Argentina | *Macruronus magellanicus* | Patagonian Shelf | INIDEP | 151,773.0 | 2006 | 2.15 | 0.60 | 1 | S2 |
| Patagonian toothfish Macquarie Island | *Dissostichus eleginoides* | Subantarctic High Seas | CSIRO | 68.7 | 2010 | 2.26 | 0.19 | 1 | S2 |
| Petrale sole Northern Pacific Coast | *Eopsetta jordani* | California Current | NWFSC | 1,838.7 | 2005 | 1.87 | 1.26 | 1 | S2 |
| Petrale sole Southern Pacific Coast | *Eopsetta jordani* | California Current | NWFSC | 514.1 | 2005 | 1.13 | 0.61 | 1 | S2 |
| Pollock NAFO-4VWX5Zc | *Pollachius virens* | Northeast U.S. Shelf | DFO | 2,504.0 | 2006 | 0.56 | 0.30 | 2 | S2 |
| Red Grouper Gulf of Mexico | *Epinephelus morio* | Gulf of Mexico | SEFSC | 3,709.7 | 2005 | 1.27 | 0.73 | 1 | S2 |
| Red king crab Bristol Bay | *Paralithodes camtschaticus* | East Bering Sea | AFSC | 10,534.7 | 2007 | 1.11 | 0.31 | 1 | S2 |
| Red rock lobster New Zealand CRA4 | *Jasus edwardsii* | New Zealand Shelf | NZMF | 641.2 | 2005 | 0.67 | 1.33 | 2 | S2 |
| Red rock lobster New Zealand CRA7 | *Jasus edwardsii* | New Zealand Shelf | NZMF | 100.7 | 2005 | 0.73 | 0.43 | 2 | S2 |
| Red rock lobster New Zealand CRA8 | *Jasus edwardsii* | New Zealand Shelf | NZMF | 629.6 | 2005 | 0.69 | 0.49 | 2 | S2 |
| Redfish species NAFO 3LN | *Sebastes spp* | Newfoundland-Labrador Shelf | NAFO | 1,664.0 | 2007 | 1.88 | 0.04 | 1 | S2 |
| Rougheye rockfish Gulf of Alaska | *Sebastes aleutianus* | Gulf of Alaska | AFSC | 425.0 | 2007 | 1.64 | 0.26 | 1 | S2 |
| Sablefish Pacific Coast | *Anoplopoma fimbria* | California Current | NWFSC | 4,629.6 | 2007 | 2.13 | 0.69 | 1 | S2 |
| Saithe Faroe Plateau | *Pollachius virens* | Faroe Plateau | ICES | 62,539.0 | 2010 | 1.07 | 1.35 | 3 | S40 |
| Saithe Iceland | *Pollachius virens* | Iceland Shelf | ICES | 53,853.0 | 2010 | 0.50 | 1.32 | 3 | S40 |
| Sandeel Central Eastern North Sea SA3 | *Ammodytes marinus* | North Sea | ICES | 78,067.0 | 2010 | 0.66 | 0.32 | 2 | S33 |
| Sandeel North Sea Dogger Bank SA1 | *Ammodytes marinus* | North Sea | ICES | 285,794.0 | 2010 | 1.86 | 0.28 | 2 | S33 |
| Sandeel South Eastern North Sea SA2 | *Ammodytes marinus* | North Sea | ICES | 30,530.0 | 2010 | 1.71 | 0.16 | 2 | S33 |
| Sardine South Africa | *Sardinops sagax* | Benguela Current | MARAM | 217,138.0 | 2006 | 0.75 | 0.55 | 2 | S2 |
| School whiting Southeast Australia | *Sillago flindersi* | Southeast Australian Shelf | CSIRO | 1,523.5 | 2007 | 0.66 | 0.82 | 2 | S2 |
| Sea scallop Georges Bank | *Placopecten magellanicus* | Northeast U.S. Shelf | NEFSC | 17,286.0 | 2006 | 1.59 | 0.78 | 2 | S2 |
| Sea scallop Mid-Atlantic Coast | *Placopecten magellanicus* | Northeast U.S. Shelf | NEFSC | 8,819.0 | 2006 | 1.00 | 0.36 | 2 | S2 |
| Silverfish Southeast Australia | *Seriolella punctata* | Southeast Australian Shelf | CSIRO | 2,585.3 | 2006 | 1.03 | 0.79 | 2 | S2 |
| Skipjack tuna Eastern Atlantic | *Katsuwonus pelamis* | Atlantic High Seas | ICCAT | 115,723.3 | 2006 | 1.71 | 0.27 | 2 | S2 |
| Skipjack tuna Western Atlantic | *Katsuwonus pelamis* | Atlantic High Seas | ICCAT | 26,453.6 | 2006 | 1.72 | 0.32 | 2 | S2 |
| Smooth oreo Chatham Rise | *Pseudocyttus maculatus* | New Zealand Shelf | NZMF | 5,946.0 | 2006 | 2.25 | 0.38 | 1 | S2 |
| Snow crab Bering Sea | *Chionoecetes opilio* | East Bering Sea | AFSC | 34,956.5 | 2008 | 0.55 | 1.49 | 2 | S2 |
| Southern blue whiting Campbell Island | *Micromesistius australis* | New Zealand Shelf | NZMF | 19,763.0 | 2006 | 1.15 | 0.92 | 1 | S2 |
| Southern Bluefin Tuna | *Thunnus maccoyii* | Pacific High Seas | CCSBT | 11,447.0 | 2009 | 0.19 | 1.73 | 1 | S49 |
| Southern hake Chatham Rise | *Merluccius australis* | New Zealand Shelf | NZMF | 600.0 | 2006 | 1.77 | 0.12 | 1 | S2 |
| Southern hake Sub-Antarctic | *Merluccius australis* | New Zealand Shelf | NZMF | 2,400.0 | 2007 | 2.91 | 0.11 | 1 | S2 |
| Southern spiny lobster South Africa | *Palinurus gilchristi* | Agulhas Current | MARAM | 363.0 | 2008 | 0.51 | 1.50 | 2 | S2 |
| Spanish mackerel Southern Atlantic | *Scomberomorus maculatus* | Southeast U.S. Shelf | SEFSC | 2,215.5 | 2007 | 0.47 | 0.91 | 1 | S2 |
| Starry flounder Northern Pacific Coast | *Platichthys stellatus* | California Current | SWFSC | 104.0 | 2005 | 1.10 | 0.33 | 1 | S2 |
| Starry flounder Southern Pacific Coast | *Platichthys stellatus* | California Current | SWFSC | 46.0 | 2005 | 1.55 | 0.12 | 1 | S2 |
| Striped marlin NE Pacific Ocean | *Kajikia audax* | Pacific High Seas | IATTC | 1,296.0 | 2008 | 1.27 | 0.35 | 1 | S50 |
| Swordfish Indian Ocean | *Xiphias gladius* | Indian Ocean | IOTC | 21,451.0 | 2009 | 1.59 | 0.43 | 1 | S51 |
| Swordfish Mediterranean Sea | *Xiphias gladius* | Mediterranean Sea | ICCAT | 14,600.0 | 2006 | 0.94 | 1.27 | 2 | S52 |
| Swordfish South Atlantic | *Xiphias gladius* | Atlantic High Seas | ICCAT | 12,690.0 | 2009 | 1.04 | 0.75 | 1 | S53 |
| Tanner crab BSAI | *Chionoecetes bairdi* | East Bering Sea | AFSC | 3,991.6 | 2007 | 0.79 | 0.15 | 1 | S2 |
| Tautog Rhode Island | *Tautoga onitis* | Northeast U.S. Shelf | RIDEM | 203.0 | 2006 | 0.84 | 0.59 | 2 | S2 |
| Tiger flathead Southeast Australia | *N. richardsoni* | Southeast Australian Shelf | CSIRO | 3,199.2 | 2006 | 1.99 | 1.03 | 2 | S2 |
| Tilefish Mid-Atlantic coast | *L. chamaeleonticeps* | Northeast U.S. Shelf | SEFSC | 868.0 | 2005 | 0.72 | 0.61 | 1 | S2 |
| Trevally New Zealand Areas TRE 7 | *Pseudocaranx dentex* | New Zealand Shelf | NZMF | 2,454.0 | 2005 | 1.44 | 0.83 | 1 | S2 |
| Vermilion snapper Southern Atlantic | *Rhomboplites aurorubens* | Southeast U.S. Shelf | SEFSC | 460.6 | 2007 | 0.80 | 1.27 | 1 | S2 |
| White hake Georges Bank | *Urophycis tenuis* | Northeast U.S. Shelf | NEFSC | 2,207.2 | 2007 | 0.35 | 0.80 | 1 | S2 |
| Whiting ICES VIIe-k | *Merlangius merlangus* | Celtic-Biscay Shelf | ICES | 9,500.0 | 2010 | 0.92 | 0.52 | 2 | S2 |
| Widow rockfish Pacific Coast | *Sebastes entomelas* | California Current | NWFSC | 281.0 | 2006 | 0.91 | 0.05 | 2 | S2 |
| Winter Flounder NAFO 5Z | *P. americanus* | Northeast U.S. Shelf | NEFSC | 990.4 | 2006 | 0.28 | 0.25 | 2 | S2 |
| Winter flounder Rhode Island | *P. americanus* | Northeast U.S. Shelf | RIDEM | 496.0 | 2006 | 0.23 | 2.02 | 2 | S2 |
| Winter Flounder S New England | *P. americanus* | Northeast U.S. Shelf | NEFSC | 1,857.0 | 2007 | 0.09 | 1.10 | 2 | S2 |
| Witch Flounder NAFO 5Y | *Glyptocephalus cynoglossus* | Northeast U.S. Shelf | NEFSC | 1,171.6 | 2007 | 0.30 | 1.45 | 1 | S2 |
| Yelloweye rockfish Pacific Coast | *Sebastes ruberrimus* | California Current | NWFSC | 14.4 | 2006 | 1.11 | 0.65 | 2 | S2 |
| Yellowfin tuna Atlantic | *Thunnus albacares* | Atlantic High Seas | ICCAT | 108,623.1 | 2006 | 1.07 | 0.81 | 2 | S54 |
| Yellowfin tuna Central Western Pacific | *Thunnus albacares* | Pacific High Seas | SPC | 413,417.9 | 2005 | 1.29 | 0.80 | 2 | S55 |
| Yellowfin tuna Eastern Pacific | *Thunnus albacares* | Pacific High Seas | IATTC | 255,923.0 | 2010 | 0.71 | 1.13 | 1 | S56 |
| Yellowfin tuna Indian Ocean | *Thunnus albacares* | Indian Ocean | IOTC | 325,854.0 | 2009 | 1.02 | 1.15 | 1 | S57 |
| Yellowtail flounder Gulf of Maine | *Limanda ferruginea* | Northeast U.S. Shelf | NEFSC | 627.0 | 2007 | 0.25 | 1.73 | 1 | S2 |
| Yellowtail flounder Georges Bank | *Limanda ferruginea* | Northeast U.S. Shelf | NEFSC | 1,686.0 | 2007 | 0.22 | 1.14 | 1 | S2 |
| Yellowtail Flounder New England | *Limanda ferruginea* | Northeast U.S. Shelf | NEFSC | 396.0 | 2007 | 0.13 | 1.61 | 1 | S2 |
| Yellowtail rockfish Pacific Coast | *Sebastes flavidus* | California Current | NWFSC | 1,613.5 | 2005 | 1.36 | 0.51 | 2 | S2 |
|  |  |  |  |  |  |  |  |  |  |
