# Supplementary material for: Eco-Label Conveys Reliable Information on Fish Stock Health to Seafood Consumers
Source: PLoS One. 2012 Aug 21;7(8):e43765. doi: 10.1371/journal.pone.0043765 (PMC3424161; doi:10.1371/journal.pone.0043765)
Supplement: Table S3 — Median (±SE) biomass and exploitation rates relative to their targets and differences among certified, uncertified and not-recommended stocks. *denotes statistical significance (*P<0.05; **P<0.005). (DOCX) [file pone.0043765.s003.docx]

**Table S3.** Median (± SE) biomass and exploitation rates relative to their targets and differences among certified, uncertified and not-recommended stocks. *denotes statistical significance (**P* < 0.05; ***P* < 0.005).

|  | Certified^+^ | Uncertified | Not recommended | ∆_(Certified-Uncertified)_ | ∆_(Certified- Not recommended)_ | ∆_(Uncertified-Not recommended)_ |
| --- | --- | --- | --- | --- | --- | --- |
| Number of stocks analyzed | 45 | 179 | 25 |  |  |  |
| *B_current_*/*B_MSY_* | 1.25 (0.22) | 0.87 (0.13) | 0.48 (0.09) | 0.38** | 0.77** | 0.39** |
| *u_current_*/*u_MSY_* | 0.67 (0.09) | 0.73 (0.12) | 0.92 (0.36) | -0.06 | -0.25** | -0.19* |
| % stocks *B_current_*> *B_MSY_* | 74 | 44 | 16 | 30* | 58** | 28* |
| % stocks with *u_current_*< *u_MSY_* | 82 | 65 | 52 | 17* | 30** | 13 |
| % stocks with *B_current_*< 0.5*B_MSY_* | 7 | 27 | 52 | -20* | -45** | -25* |
| % stocks with *B_current_* > 1.3*B_MSY_* | 49 | 29 | 4 | 20* | 45** | 25* |
