# Supplementary material for: Eco-Label Conveys Reliable Information on Fish Stock Health to Seafood Consumers
Source: PLoS One. 2012 Aug 21;7(8):e43765. doi: 10.1371/journal.pone.0043765 (PMC3424161; doi:10.1371/journal.pone.0043765)
Supplement: Table S4 — Median biomass and exploitation rates relative to their targets and differences among certified, uncertified and not-recommended stocks for those fisheries with both B MSY and F MSY available from stock assessments (DOCX) [file pone.0043765.s004.docx]

**Table S4.** Median biomass and exploitation rates relative to their targets and differences among certified, uncertified and not-recommended stocks for those fisheries with both *B*_MSY_ and *F*_MSY_ or *u*_MSY_ available from stock assessments.

|  | Certified | Uncertified | Not recommended | ∆_(Certified-Uncertified)_ | ∆_(Certified- Not recommended)_ | ∆_(Uncertified-Not recommended)_ |
| --- | --- | --- | --- | --- | --- | --- |
| *B_current_*/*B_MSY_* | 1.6 | 1.03 | 0.48 | 0.57 | 1.12 | 0.55 |
| *u_current_*/*u_MSY_* | 0.45 | 0.61 | 0.92 | -0.16 | -0.5 | -0.31 |
| % stocks *B_current_*> *B_MSY_* | 79 | 52 | 16 | 27 | 63 | 36 |
| % stocks with *u_current_*< *u_MSY_* | 93 | 73 | 52 | 20 | 41 | 21 |
| % stocks with *B_current_*< 0.5*B_MSY_* | 0 | 23 | 51 | -23 | -51 | -28 |
| % stocks with *B_current_* > 1.3*B_MSY_* | 59 | 36 | 4 | 23 | 55 | 32 |
