# Supplementary material for: Eco-Label Conveys Reliable Information on Fish Stock Health to Seafood Consumers
Source: PLoS One. 2012 Aug 21;7(8):e43765. doi: 10.1371/journal.pone.0043765 (PMC3424161; doi:10.1371/journal.pone.0043765)
Supplement: Table S5 — Results of test for difference in mean B/B MSY over time between certified and uncertified stocks. *denotes significance (*P<0.05; **P<0.005). (DOCX) [file pone.0043765.s005.docx]

**Table S5.** Results of test for difference in mean *B/B*_MSY_ over time between certified and uncertified stocks. *denotes significance (**P* < 0.05; ***P* < 0.005).

|  | Coefficient Estimate | Standard Error | p-value |
| --- | --- | --- | --- |
| (Intercept) | 0.0215 | 0.0032 | 1e-11** |
| Certified | 0.0145 | 0.0057 | 0.0115** |
| First-order lag | 1.3685 | 0.0105 | <2e-16** |
| Second-order lag | -0.3924 | 0.0104 | <2e-16** |
|  |  |  |  |
