# Supplementary material for: Eco-Label Conveys Reliable Information on Fish Stock Health to Seafood Consumers
Source: PLoS One. 2012 Aug 21;7(8):e43765. doi: 10.1371/journal.pone.0043765 (PMC3424161; doi:10.1371/journal.pone.0043765)
Supplement: Text S1 — This supporting information file includes expanded descriptions of methods used, additional results and model outputs and supporting references. (DOCX) [file pone.0043765.s006.docx]

**Supporting Information**

**Methods**

***Data compilation***

We compiled time series of catch data, fishing mortality rates, and model estimates of biomass to assess the status of 45 fish stocks currently targeted by MSC-certified fisheries (Table S1) and 179 uncertified fish stocks (Table S2). Additionally, for each stock we collected the biomass (*B*_MSY_) and exploitation rate (*u*_MSY_) or fishing mortality (*F*_MSY_) that result in Maximum Sustainable Yield (MSY). When available, we used MSY reference points estimated within a stock assessment model, and collected biomass time series that corresponded to the same units as *B*_MSY_. If these reference points were not reported by the stock assessment agency or scientific advisory body, we fit a surplus production model to time series of biomass and total catch to estimate these reference points (see description of surplus production fitting method below). If multiple estimates of biomass were reported (e.g., spawning biomass) total biomass estimates were used.

Data for MSC-certified stocks were collected from the most recent stock assessment reports or from personal communication with stock assessment scientists. The MSC defines the unit of certification for a fishery based on several variables (e.g., gear type, organizational framework, port of landing). As a result, a single stock may be fished by multiple certified fisheries or, alternatively, one fishery may target different stocks. Thus, the 45 stocks we analysed included 82 (62%) certified fisheries. Three types of MSC-certified fisheries were not included in our dataset, as follows: (1) fisheries that are assessed under different approaches than single-species MSY (for example salmon and invertebrates); (2) those stocks without MSY reference points and without time series from which to obtain such reference points; and (3) those certified fisheries managed under traditional or informal management schemes and/or categorized as data limited (without MSY reference points; Table S1). These fisheries are scored using MSC’s risk-based framework (RBF) [1], which takes into account a wide range of metrics including the resilience of the target species, the type of fishing method employed and the decision-making process of the management body to assess the sustainability of the fishery. The RBF was developed to increase the accessibility to the MSC program of sustainable small-scale fisheries, data-limited fisheries or fisheries in developing countries [1].

The majority of the data for uncertified fish stocks was sourced from the RAM Legacy Stock Assessment Database [2]. This database represents almost all of the world’s fish stocks for which assessments are regularly conducted. As such it is often geographically limited to stocks exploited by developed countries with well-established fisheries management agencies and large, industrial-scale fisheries, and so our data set is largely comprised of stocks that meet these characteristics, making them comparable with certified stocks. We used all stocks in the RAM Legacy database that met our data collection requirements (outlined above). For some stocks in the RAM Legacy database more recent stock assessments were available. We updated the data whenever possible (see Table S2 for a list of sources), and discarded assessments older than 2005.

In order to assess whether only sustainable fisheries were applying for MSC certification and to further evaluate the performance of MSC’s certification standards and the MSC screening process, we compared certified stocks with those that went through the pre-assessment process and were recommended not to pursue full certification. Pre-assessment is a confidential process used to assess a fishery’s ability to meet MSC’s standards. Fisheries receiving a recommendation not to go forward will usually withdraw before entering the full, public certification process. We compiled a subset of 25 non-recommended stocks from available MSC proprietary and confidential information on stocks that had undergone pre-assessment and were not recommended to proceed into full assessment and for which information on biomass reference points were available. We confined our subset to those stocks that showed weaknesses with respect to MSC’s standards for biological stock status (Principle 1).

***Assessing stock status***

We assessed the status of each stock based on: (1) the current biomass relative to the biomass that would produce MSY (*B*_current_/ *B*_MSY_), and (2) the current exploitation rate or fishing mortality relative to the exploitation rate that would produce MSY (*u*_current_ / *u*_MSY_). When stock assessments provided estimates of instantaneous fishing mortality (*F*), those were used to calculate *F*_current_/*F*_MSY_. Whenever possible we used the MSY reference points estimated by the management agency conducting the stock assessment (30 certified and 136 non-certified stocks). For fish stocks under the International Council for the Exploration of the Sea (ICES), the assessments for many stocks report *F*_MSY_ as the only reference point. For those cases (12 certified and 26 non-certified stocks), we used a combination of the *B*_MSY_ estimated from the surplus production models and *F*_MSY_ estimated by the scientific advisory body (see Table S1 for the method used for certified fisheries, and Table S2 for uncertified fisheries).

If no MSY reference points were available, we estimated MSY from a surplus production model [3]. To summarize, surplus production (*P*) in year *t* is given by the following equation:


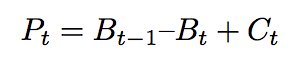


(1)

where *B* is biomass and *C* is total catch. We fit a dynamic Schaefer production model, which uses a logistic growth function to update biomass in each year, to time series with ≥20 years of biomass estimates and catch data. The surplus production predicted the Schaefer model in year *t* is given by the following equation:


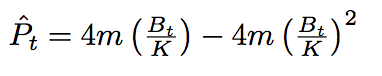
,

(2)

where *m* is the Maximum Sustainable Yield, and *K* is the unfished biomass. These parameters (*m* and *K*) were estimated using maximum likelihood in AD Model Builder [4]. We assumed that the residuals were normally distributed [3]. The Schaefer model assumes a symmetric relationship between biomass and yield, so production is maximized at 0.5*K*, providing an estimate of *B*_MSY_. The exploitation rate that results in MSY (*u*_MSY_) is defined as the yield (*m*) divided by the available biomass at this yield, or *m*/*B*_MSY_. Stocks with poor fits or unreasonable estimates were discarded.

Even though the concept of MSY has been extensively used in fisheries science and in internationally agreed target or limit reference points, it is not uniformly defined or estimated. Numeric estimates of *B*_MSY_ and *u*_MSY_ are dependent on the vulnerability of different sizes or ages of fish to the fishing gear, on whether total biomass or spawning biomass are used to define *B*_MSY_, and on the model or method used for estimation [3]. For the latter, MSY reference points are particularly sensitive to the underlying assumptions in the stock-recruitment relationships. In order to minimize uncertainty related to stock-recruitment relationships and to avoid assumptions on steepness, we fit the described surplus production models against total biomass.

***Data analysis***

We examined the status of three groups of fisheries (certified, uncertified, and non-recommended) by plotting *B*/*B*_MSY_ vs. *u*/*u*_MSY_ or *F*/*F*_MSY_. The current (or most recent) status for each of the 45 certified stocks are plotted in Fig. 1A. The 179 uncertified stocks are plotted in Fig. 1B, and the non-recommended stocks are plotted in Fig. 1C. A kernel density smoothing function was used to describe the probability of occurrence in each quadrant (Fig. 1A, 1B, and 1C).

To determine whether *B*/ *B*_MSY_ is significantly different between groups we used re-sampling inference, which allows us to assess how often a difference of the observed magnitude or larger would arise by chance. We combined our certified and uncertified datasets for *B* /*B*_MSY_ (*n* = 224) and randomly drew (without replacement) a sample of 45 relative biomass points. We then calculated the average relative biomass of the selected data points as well as the average relative biomass of the unselected data points, and calculated the difference between the two. We repeated this process 100,000 times in order to calculate the probability of observing a greater difference in *B*/ *B*_MSY_ between two randomly observed data groupings. The same process was repeated to determine the probability of observing significant differences in *u* / *u*_MSY_ between groups.

We conducted three additional analyses using re-sampling inference. The first tested whether certified fisheries were more likely to have *B* > *B*_MSY_ than uncertified fisheries. The second tested whether certified fisheries were more likely to have *u* < *u*_MSY_. We also ran each of these tests using *B*_MSY_ and *u*_MSY_ as strict cut-offs. The third tested whether the number of uncertified stocks that are below 0.5*B*_MSY_ is significantly different from the number of certified stocks at the same biomass levels.

Because fish stocks can be highly variable from year to year, we examined the long-term performance of certified and uncertified stocks in relation to *B_MSY_* (Fig. 2). The available time series data (165 uncertified stocks and 31 certified stocks) was fit to the following model to test for a difference in the conditional mean of each group:


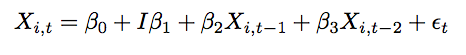


(3)

where *X* is the ratio of *B*/*B*_MSY_ for stock *i* in time period *t*, and *I* is an indicator variable describing whether stock *i* is certified at any point in the observed time trajectory. Estimation of parameters using Ordinary Least Squares assumes that the error term is uncorrelated with the regressors. Because time series observations are not temporally independent, we included two autoregressive lag parameters in our linear model. Autoregressive models can correct for any biases that may result from correlation between the error term and one or more regressors. To select the appropriate number of lags, an autoregressive model was fit to each stock time series and the order was selected using Akaike’s Information Criterion (AIC; > 90% of all stocks had an order of two or less).

**Results**

The median *B*/*B*_MSY_ was 1.25 for certified stocks (*n* = 45) and 0.87 for uncertified stocks (*n* =179). We found the difference (0.38) in *B*/*B*_MSY_ between the two groups to be statistically significant (*P* < 0.05; see Supporting Table 3 for all statistical results). A similar resampling calculation was made to determine whether *u*/*u*_MSY_ for certified stocks is lower than *u*/*u*_MSY_ for uncertified stocks. In this case, the median was 0.67 for certified stocks and 0.73 for uncertified stocks, but the difference (-0.06) was not statistically significant (*P* = 0.19).

Some 74% of certified fisheries had a biomass above *B*_MSY_ (i.e. *B* > *B*_MSY_), compared with 44% of uncertified fisheries. We found significantly more certified fisheries above *B*_MSY_ than expected by random selection from all fisheries, compared with the uncertified fisheries (*P* < 0.05). In addition, 82% of certified fisheries vs. 65% of uncertified stocks had exploitation rates that would maintain the stock around *B*_MSY_ or allow rebuilding to *B*_MSY_ (i.e., *u* < *u*_MSY_). When we compared the difference in these percentages with the difference between randomly chosen groups of stocks, certified stocks had a significantly higher chance of being 17% better than uncertified stocks in this metric (*P* < 0.05). We also observed that 3 of 45 stocks (7%) were below 0.5*B*_MSY_ (excluding the suspended Iberian sardine stock) whereas 49 of 179 uncertified stocks (27%) were below 0.5*B*_MSY_. We tested the probability of observing a greater difference in the number of overfished stocks in each group. Significantly fewer certified stocks were below *B*_MSY_ compared to uncertified stocks (*P* < 0.05). Finally, when comparing the status of the certified, uncertified, and non-recommended stocks by using only those fisheries with stock assessment and reference points defined by the management agencies or scientific advisory bodies, we observed similar trends, and differences among categories (Table S4).

The median biomass in relation to *B*_MSY_ for certified and uncertified stocks is shown in Figure 2. Certified stocks had significantly higher *B*/*B*_MSY_ ratios than uncertified stocks (Table S5). Given that this analysis is conducted with time series data that include observations prior to the creation of MSC, we do not draw causal conclusions regarding the effect of certification. When we performed the same test on a subset of the observations prior to 1998 the same effect was observed. However, this test does indicate that the stocks that have been certified by MSC are outperforming other stocks in standing stock biomass, an important indicator of long-term stock sustainability.

**Supporting acknowledgements**

S.R.V., J.C.D., A.E.L., R.L.S., S.S, S.J.T and N.E.W thank the Henry Luce Foundation’s support of UCSB’s Environmental Science to Solutions Fellowship Program, which provided needed funding for our working group “Evaluating the sustainability of MSC-certified fisheries” at the National Centre for Ecological Analysis and Synthesis, a Centre funded by NSF (Grant #EF-0553768), the University of California, Santa Barbara, and the State of California. S.R.V. acknowledges support by the National Science Foundation Graduate Research Fellowship under Grant No. DGE-0707430. J.C.D. would like to thank Fulbright and the Chilean government's Comición Nacional de Ciencia y Tecnología for his graduate student fellowship. O.D. acknowledges support by the Pew Charitable Trusts. R.L.S. thanks the Department of Defense (DoD) through the National Defense Science & Engineering Graduate Fellowship (NDSEG) Program for financial support. J.T.T. acknowledges support from the University of Washington, the NMFS-Sea Grant Population Dynamics Fellowship (NA09OAR4170120), and a NMFS groundfish project grant to the University of Washington.

**Supporting References**

S1. Marine Stewardship Council (2010) Fisheries Assessment Methodology v.2, [http://www.msc.org/documents/scheme-documents/msc-scheme-requirements/methodologies/Fisheries_Assessment_Methodology.pdf](http://www.msc.org/documents/scheme-documents/msc-scheme-requirements/methodologies/Fisheries_Assessment_Methodology.pdf%20) .

S2. Ricard D, Minto C, Jensen OP, Baum JK (2011) Examining the knowledge base and status of commercially exploited marine species with the RAM Legacy Stock Assessment Database. *Fish and Fisheries* 1–19. DOI: 10.1111/j.1467-2979.2011.00435.x.

S3. Worm B, Hilborn R, Baum JK, Branch TA, Collie JS, et al. (2009) Rebuilding global fisheries. Science 325: 578-585.

S4. ADMB Project (2010) *AD Model Builder: Automatic Differentiation Model Builder.* Developed by David Fournier and freely available from admb-project.org.

S5. ICCAT, *Report of the 2009 Atlantic Swordfish Stock Assessment Session*. Collect. Vol. Sci. Pap. ICCAT 65, 1–123 (Collect. Vol. Sci. Pap. ICCAT: Madrid, 2010).

S6. ICES (2011) *Report of the Baltic Fisheries Assessment Working Group (WGBFAS).* ICES CM 2011/ACOM:11, 1–824.

S7. ICES (2011) *Report of the Arctic Fisheries Working Group (AFWG).* ICES CM 2011/ACOM:05, 1–678.

S8. Rademeyer R, Butterworth D, *Final Set of Candidate management Procedures for the South African hake resource*. FISHERIES/2010/October/SWG-DEM/53, 1–7 (Demersal Working Group Report, 2011).

S9. Vega VA, Treviño GE, Espinoza CG, Zuñiga PLC. *Evaluación de la pesquería de langosta roja (Panulirus interruptus) en la región centro occidental de la península de Baja California, mediante modelos dinámicos de biomasa: puntos de referencia y recomendaciones de manejo*. 1–20 (Informe Técnico CRIP La Paz. INAPESCA-SAGARPA: 2010).

S10. Hare SR (2011) *Assessment of the Pacific halibut stock at the end of 2010*. International Pacific Halibut Commission 1–91, <<http://www.iphc.int/papers/sa10.pdf>>.

S11. Stewart I et al. (2011) Stock Assessment of Pacific Hake, *Merluccius productus*, (aka Whiting) in US and Canadian Waters in 2011. Joint U.S. and Canadian Hake Technical Working Group, <http://www.pacificwhiting.org/images/2011_Pacific_hake_assessment_final_SAFE_document.pdf>.

S12. Cox SP, Kronlund AR, Lacko L. 2011. Management procedures for the multi-gear sablefish (*Anoplopoma fimbria*) fishery in British Columbia, Canada. CSAP Working Paper P2010-05. <http://www.dfo-mpo.gc.ca/Csas-sccs/publications/resdocs-docrech/2011/2011_063-eng.pdf>.

S13. ICES (2011) *Report of the Working Group on Widely Distributed Stocks (WGWIDE*). ICES CM 2011/ACOM:15, 1–642.

S14. ICES (2011) *Report of the Working Group on the Assessment of Southern Shelf Stocks of Hake, Monk and Megrim (WGHMM)*. ICES CM 2011/ACOM:11, 1–625.

S15. ICES (2011) *Report of the Working Group on the Celtic Seas Ecoregion (WGCSE).* ICES CM 2011/ACOM:12, 1–1564.

S16. ICES (2010) Report of the Working Group on Anchovy and Sardine (WGANSA). ICES CM 2010/ACOM:16, 1–295.

S17. Wilderbuer TK, Nichol DG, Spencer PD (2010) *Chapter 9: Alaska Plaice.* North Pacific Fisheries Management Council Bering Sea and Aleutian Islands Stock Assessment and Fishery Evaluation Reports, <http://www.afsc.noaa.gov/REFM/docs/2010/BSAIplaice.pdf>.

S18. Wilderbuer TK, Nichol DG (2010) *Chapter 7: Northern Rock Sole.* North Pacific Fisheries Management Council Bering Sea and Aleutian Islands Stock Assessment and Fishery Evaluation Reports, http://www.afsc.noaa.gov/REFM/docs/2010/BSAIrocksole.pdf

S19. Wilderbuer TK, Nichol DG, Ianelli J (2010) *Chapter 4: Yellowfin Sole*. North Pacific Fisheries Management Council Bering Sea and Aleutian Islands Stock Assessment and Fishery Evaluation Reports, <http://www.afsc.noaa.gov/REFM/docs/2010/BSAIyfin.pdf> .

S20. Barbeaux S, Ianelli J, Gaichas S, Wilkins M (2010) *Chapter 1A: Assessment of the Pollock stock in the Aleutian Islands,* <http://www.afsc.noaa.gov/REFM/docs/2010/AIpollock.pdf>.

S21. Ianelli JN et al*.* (2010) *Chapter 1: Assessment of the walleye pollock stock in the Eastern Bering Sea.* North Pacific Fisheries Management Council Bering Sea and Aleutian Islands Stock Assessment and Fishery Evaluation Reports, <http://www.afsc.noaa.gov/REFM/docs/2010/EBSpollock.pdf>.

S22. Hanselman DH, Lunsford CR, Rodgveller CJ (2010) *Chapter 3: Assessment of the Sablefish stock in Alaska*. North Pacific Fisheries Management Council Bering Sea and Aleutian Islands Stock Assessment and Fishery Evaluation Reports, <http://www.afsc.noaa.gov/REFM/docs/2010/BSAIsablefish.pdf>.

S23. Wilderbuer TK, Nichol DG, Aydin K. (2010) *Chapter 6: Arrowtooth Flounder*. North Pacific Fisheries Management Council Bering Sea and Aleutian Islands Stock Assessment and Fishery Evaluation Reports, <http://www.afsc.noaa.gov/REFM/docs/2010/BSAIatf.pdf>.

S24. Stockhausen WT, Nichol D, Lauth R, Wilkins M (2010) *Chapter 8: Assessment of the Flathead Sole Stock in the Bering Sea and Aleutian Islands.* North Pacific Fisheries Management Council Bering Sea and Aleutian Islands Stock Assessment and Fishery Evaluation Reports, <http://www.afsc.noaa.gov/REFM/docs/2010/BSAIflathead.pdf>.

S25. Thompson GG, Ianelli JN, Lauth RR (2010) *Chapter 2: Assessment of the Pacific Cod Stock in the Eastern Bering Sea and Aleutian Islands Area*. North Pacific Fisheries Management Council Bering Sea and Aleutian Islands Stock Assessment and Fishery Evaluation Reports, <http://www.afsc.noaa.gov/REFM/docs/2010/BSAIpcod.pdf>.

S26. Dorn M et al. (2010) *Chapter 1: Assessment of the Walleye Pollock Stock in the Gulf of Alaska.* North Pacific Fisheries Management Council Gulf of Alaska Stock Assessment and Fishery Evaluation Reports, <http://www.afsc.noaa.gov/REFM/docs/2010/GOApollock.pdf> .

S27. Thompson GG, Ianelli JN, Wilkins ME (2010) *Chapter 2: Assessment of the Pacific Cod Stock in the Gulf of Alaska*. North Pacific Fisheries Management Council Gulf of Alaska Stock Assessment and Fishery Evaluation Reports, <http://www.afsc.noaa.gov/REFM/docs/2010/GOApcod.pdf>.

S28. Turnock BJ, Wilderbuer TK (2009) *Chapter 7: Gulf of Alaska Arrowtooth Flounder Stock Assessment*. North Pacific Fisheries Management Council Gulf of Alaska Stock Assessment and Fishery Evaluation Reports, <http://www.afsc.noaa.gov/refm/docs/2009/GOAatf.pdf>.

S29. Stockhausen WT, Wilkins ME, Martin MH (2009) *Chapter 8: Assessment of the Flathead Sole Stock in the Gulf of Alaska.* North Pacific Fisheries Management Council Gulf of Alaska Stock Assessment and Fishery Evaluation Reports, <http://www.afsc.noaa.gov/refm/docs/2009/GOAflathead.pdf>.

S30. Stockhausen WT, Wilkins ME, Martin MH (2009) *Chapter 6: Assessment of the Rex Sole Stock in the Gulf of Alaska.* North Pacific Fisheries Management Council Gulf of Alaska Stock Assessment and Fishery Evaluation Reports, <http://www.afsc.noaa.gov/refm/docs/2009/GOArex.pdf> .

S31. New Zealand Ministry of Fisheries (2011) *Report from the Fisheries Assessment Plenary: stock assessments and yield estimates*, [http://fs.fish.govt.nz/Doc/22702/FINAL Part 1 ver2.pdf](http://fs.fish.govt.nz/Doc/22702/FINAL Part%201%20ver2.pdf).

S32. ICES (2010) *Report of the Working Group on the Assessment of Demersal Stocks in the North Sea and Skagerrak (WGNSSK).* ICES CM 2010/ACOM: 13, 1–1058.

S33. ICES (2011) *Report of the Herring Assessment Working Group for the Area South of 62^o^N (HAWG).* ICES CM 2011/ACOM:06, 1–749.

S34. ICES (2011) *Report of the Working Group on the Assessment of Demersal Stocks in the North Sea and Skagerrak (WGNSSK).* ICES CM 2011/ACOM: 13, 1–1197.

S35. Parsons DM, Brodie WB, Morgan MJ, Power D (2008) *The 2008 Assessment of the Grand Bank Yellowtail Flounder Stock, NAFO Divisions 3LNO*. NAFO SCR Doc. 08/45, 1–50 (Northwest Atlantic Fisheries Organization, DFO).

S36. Department of Fisheries (2010) *Assessment of the Status of Division 4X5Y Haddock in 2009*. DFO Can Sci. Advis. Sec. Sci. Advis. Rep. 2010/005.

S37. TRAC (2010) *Eastern Georges Bank Cod* (5Zjm; 551, 552, 561, 562). Guidance Document 2010/01, 1–10 (TRAC Status Report 2010/01).

S38. Hoyle S, Davies N (2009) *Stock Assessment of Albacore Tuna in the South Pacific Ocean. Western and Central Pacific Fisheries Commission* WCPFC-SC5-2009/SA-WP-6, 1–134 (Western and Central Pacific Fisheries Commission: Port Vila, Vanuatu).

S39. Hoyle S, Kleiber P, Davies N, Harley S, Hampton J (2010) *Stock Assessment of Skipjack Tuna in the Western and Central Pacific Ocean.* Western and Central Pacific Fisheries Commission WCPFC-SC6-2010/SA-WP-10 rev.1, 1–118 (Western and Central Pacific Fisheries Commission: Nuku'alofa, Tonga).

S40. CCAMLR (2011) Fishery Report: *Dissostichus Eleginoides* South Georgia (Subarea 48.3), http://www.ccamlr.org/pu/e/e_pubs/fr/11/appG.pdf

S41. ICES (2011; 2010) *Report of the North-Western Working Group (NWWG).* 07, ICES CM 2011 ACOM, 1–975.

S42. ICCAT (2010) *Report of the 2010 ICCAT bigeye tuna stock assessment session,* <http://www.iccat.int/Documents/Meetings/Docs/2010_BET_Assessment_REP_ENG.pdf>.

S43. Aires-da-Silva A, Maunder MN (2011) *Status of bigeye tuna in the Eastern Pacific Ocean in 2010 and outlook for the future*, <http://www.iattc.org/Meetings2011/May-SAC-Shark/PDFfiles/SAC-02-07-BET-assessment-2010.pdf>.

S44. Langley A, Hampton J, Kleiber P, Hoyle S (2008) *Stock assessment of bigeye tuna in the Western and Central Pacific Ocean, including an analysis of management options*. WCPFC-SC4-2008/SA-WP Rev.1.

S45. ICCAT (2010) *Report of the 2010 Atlantic bluefin tuna stock assessment session*, <http://www.iccat.int/Documents/Meetings/Docs/2010_BFT_ASSESS_REP_ENG.pdf>.

S46. ICCAT (2008) *Report of the 2008 Atlantic bluefin tuna stock assessment session*. <http://www.iccat.int/Documents/Meetings/Docs/2008_BFT_STOCK_ASSESS_REP.pdf>.

S47. ICES (2010) *Report of the Working Group on the Celtic Seas Ecoregion (WGCSE).* ICES CM 2010/ACOM:12, 1–1424.

S48. ICES (2009) *Report of the Working Group on the Celtic Seas Ecoregion (WGCSE).* ICES CM 2009/ACOM:12, 1–1420.

S49. CCSBT (2010) Report of the Fifteenth Meeting of the Scientific Committee, <http://www.ccsbt.org/userfiles/file/docs_english/meetings/meeting_reports/ccsbt_17/report_of_SC15.pdf>.

S50. Hinton MG, Maunder MN (2010) *Status and trends of striped marlin in the Northeast Pacific Ocean in 2009*. Stock Assessment Report 11, 163–218 (IATTC: La Jolla, California).

S51. Kolody D, Herrera M (2011) *An age-, sex- and spatially-structured stock assessment of the Indian Ocean swordfish fishery 1950-2009, including special emphasis on the South-West region*. IOTC-2011-WPB-17.

S52. ICCAT (2010) Report of the 2010 ICCAT Mediterranean swordfish stock assessment meeting, <http://www.iccat.int/Documents/Meetings/Docs/2010_MED_SWO_ASSESS.pdf>.

S53. ICCAT (2010) *Report of the 2009 Atlantic swordfish stock assessment session, 65*, 1–123 (Collective Volume of Scientific Papers, ICCAT: Madrid, September 7 to 11, 2009).

S54. ICCAT (2008) *Report of the 2008 ICCAT yellowfin and skipjack stock assessments meeting.* SCRS/2008/016.

S55. Langley A, Hampton J, Kleiber P, Hoyle S (2007) Stock assessment of yellowfin tuna in the western and central Pacific Ocean, including an analysis of management options. WCPFC-SC3-SA SWG/WP-01.

S56. Aires-da-Silva AM, Maunder MN (2010) *Status of yellowfin tuna in the Eastern Pacific Ocean in 2010 and outlook for the future*, <http://www.iattc.org/Meetings2011/May-SAC-Shark/PDFfiles/SAC-02-06-YFT-assessment-2010.pdf> .

S57. Langley A, Herrera M, Million J (2010) Stock assessment of yellowfin tuna in the Indian Ocean using MULTIFAN-CL, <http://www.iotc.org/files/proceedings/2010/wptt/IOTC-2010-WPTT-23.pdf>.
